# Bioenergetic status modulates motor neuron vulnerability and pathogenesis in a zebrafish model of spinal muscular atrophy

**DOI:** 10.1371/journal.pgen.1006744

**Published:** 2017-04-20

**Authors:** Penelope J. Boyd, Wen-Yo Tu, Hannah K. Shorrock, Ewout J. N. Groen, Roderick N. Carter, Rachael A. Powis, Sophie R. Thomson, Derek Thomson, Laura C. Graham, Anna A. L. Motyl, Thomas M. Wishart, J. Robin Highley, Nicholas M. Morton, Thomas Becker, Catherina G. Becker, Paul R. Heath, Thomas H. Gillingwater

**Affiliations:** 1 Euan MacDonald Centre for Motor Neurone Disease Research, University of Edinburgh, Edinburgh, United Kingdom; 2 Centre for Integrative Physiology, Edinburgh Medical School: Biomedical Sciences, University of Edinburgh, Edinburgh, United Kingdom; 3 Sheffield Institute for Translation Neuroscience, University of Sheffield, Sheffield, United Kingdom; 4 University/British Heart Foundation Centre for Cardiovascular Science, University of Edinburgh, Queens Medical Research Institute, Edinburgh, United Kingdom; 5 Division of Neurobiology, Roslin Institute, University of Edinburgh, Edinburgh, United Kingdom; 6 Centre for Neuroregeneration, Edinburgh Medical School: Biomedical Sciences, University of Edinburgh, Edinburgh, United Kingdom; The Jackson Laboratory, UNITED STATES

## Abstract

Degeneration and loss of lower motor neurons is the major pathological hallmark of spinal muscular atrophy (SMA), resulting from low levels of ubiquitously-expressed survival motor neuron (SMN) protein. One remarkable, yet unresolved, feature of SMA is that not all motor neurons are equally affected, with some populations displaying a robust resistance to the disease. Here, we demonstrate that selective vulnerability of distinct motor neuron pools arises from fundamental modifications to their basal molecular profiles. Comparative gene expression profiling of motor neurons innervating the *extensor digitorum longus* (disease-resistant), *gastrocnemius* (intermediate vulnerability), and *tibialis anterior* (vulnerable) muscles in mice revealed that disease susceptibility correlates strongly with a modified bioenergetic profile. Targeting of identified bioenergetic pathways by enhancing mitochondrial biogenesis rescued motor axon defects in SMA zebrafish. Moreover, targeting of a single bioenergetic protein, phosphoglycerate kinase 1 (Pgk1), was found to modulate motor neuron vulnerability *in vivo*. Knockdown of *pgk1* alone was sufficient to partially mimic the SMA phenotype in wild-type zebrafish. Conversely, Pgk1 overexpression, or treatment with terazosin (an FDA-approved small molecule that binds and activates Pgk1), rescued motor axon phenotypes in SMA zebrafish. We conclude that global bioenergetics pathways can be therapeutically manipulated to ameliorate SMA motor neuron phenotypes *in vivo*.

## Author summary

Selective vulnerability of particular cell types is a prominent, unresolved feature of many neurodegenerative diseases. In SMA, motor neurons are the most affected cell type, but not all motor neuron pools are affected at the same rate. Some pools degenerate early in disease and others remain resistant. Understanding the mechanisms governing this selective vulnerability could identify candidates for protective modifier therapy development. Using laser capture microdissection and microarray analysis, we show that resistant motor neuron pools have a different basal bioenergetic profile compared to vulnerable motor neuron pools. We subsequently showed that defective bioenergetics is a feature in the pathogenesis of SMA, using both mouse and zebrafish SMA models. We also show that targeting of bioenergetic genes rescued motor neuron outgrowth defects in a zebrafish SMA model. In conclusion, we have identified bioenergetics as a potential protective modifier. Therapeutic targeting of these pathways could stabilize more vulnerable motor neurons which could halt or delay disease progression.

## Introduction

Spinal Muscular Atrophy (SMA) is an autosomal recessive childhood neuromuscular disorder. The disease is characterized by muscle weakness and paralysis, resulting from early neuromuscular junction (NMJ) breakdown occurring prior to a widespread loss of lower motor neurons (MN) from the ventral horn of the spinal cord [1]. SMA occurs as a consequence of reduced levels of the ubiquitously-expressed Survival Motor Neuron (SMN) protein, caused by mutations in the *Survival Motor Neuron 1* (*SMN1*) gene [2]. Complete loss of SMN protein is lethal to all cells and tissues. However, in humans a nearly identical second copy of the *SMN1* gene exists: *SMN2* [3]. Unfortunately, this gene possesses a nucleotide change (C to T) in exon 7 rendering it capable of only producing low levels of full-length SMN protein [4, 5]. SMA is usually categorized into four distinct clinical subtypes dependent on disease severity (Type I through to Type IV), where Type I represents the most severe form with death occurring within the first two years of life. A patient’s copy number of *SMN2* determines the subtype and disease severity, with a higher copy number of *SMN2* correlating with the less severe forms of the condition [6, 7]. The SMN protein has well-established, important cellular roles in the biogenesis of small nuclear ribonuclear proteins (snRNP) and pre-mRNA splicing [8–10]. However, other non-canonical functions for SMN have also been recently identified including: roles in axonal transport [11, 12], the regulation of ubiquitin homeostasis [13–15] and a contribution to endocytic pathways [16, 17].

Large alpha motor MNs are the most affected cell population in SMA [18], with a breakdown of MN inputs at the NMJ being one of the earliest pathological features of SMA, occurring prior to the onset of overt symptoms and motor neuron loss [19–24]. Given that SMN is ubiquitously expressed and required by all cells and tissues of the body, it is still unclear why MNs show a particular vulnerability in SMA [25]. Perhaps even more surprising, it has been repeatedly demonstrated that a differential sensitivity exists between pools of MNs innervating distinct muscles. In mouse models of SMA some MN pools have been shown to be readily vulnerable to degeneration, showing high levels of NMJ denervation, whereas other MN pools in the same animal can remain intact throughout the entire course of disease progression, varying considerably across muscles [21, 26]. This finding supports the hypothesis that a subset of MNs possesses unique intrinsic characteristics that protect them against degeneration [27]. What these protective properties are, however, remains to be elucidated.

The relative vulnerability of MN pools innervating anatomically distinct muscle targets has been extensively mapped in SMA mouse models by our own and other laboratories using NMJ degeneration as a direct readout of vulnerability status [26–28]. Studies of a related neurodegenerative condition, Amyotrophic Lateral Sclerosis (ALS) revealed that larger, fast-fatigable MNs were particularly susceptible, suggesting that morphological properties may be important contributors to determining selective vulnerability [29–31]. However, in SMA, the vulnerability spectrum identified across several MN pools was found to be independent from their core physical features including: position in the body of the innervated muscle, functional sub-type, nerve stump length, muscle fibre type, motor unit size, branching pattern of the MN axon, developmental synapse elimination rate, or terminal Schwann cell number [26, 28]. This strongly suggests that morphological features do not dictate the vulnerability of MN pools in SMA, and increases the likelihood that differences in the molecular properties of distinct MN pools are influencing their relative susceptibility [27]. The identification of mechanisms governing selective vulnerability in SMA has the potential to reveal novel therapeutic targets capable of directly modulating motor neuron pathology, with potential relevance for other, related neurodegenerative disorders where selective vulnerability is also present [32, 33].

In the current study, we tested the hypothesis that the molecular composition of resistant MN pools makes them better placed to deal with the cellular stresses associated with disease triggers in SMA. We present evidence showing significant differences in the basal transcriptional profile between vulnerable and disease-resistant MN pools in mice, with the most striking difference being a greater expression of mitochondrial and energy metabolism-related genes in disease-resistant MNs. Given that MNs are large, highly active cells, with elevated energy demands to maintain cellular specific functions including the firing of action potentials at pre-synaptic terminals, it is perhaps not surprising that ATP availability could be important for their resistance in disease [34]. Furthermore, it was recently shown that vulnerable fast fatigable MNs are more affected by milder changes in bioenergetic status than more resistant MNs suggesting that bioenergetics could be a key determinant of selective vulnerability in related motor neuron diseases such as amyotrophic lateral sclerosis (ALS) [35]. We therefore also show that manipulating mitochondrial and glycolytic bioenergetic pathways in *smn* morphant zebrafish can ameliorate SMA phenotypes, implicating these pathways as potential therapeutic targets.

## Results

### Comparative gene expression profiling of differentially affected motor neuron pools reveals modified molecular composition

**Fig 1:**
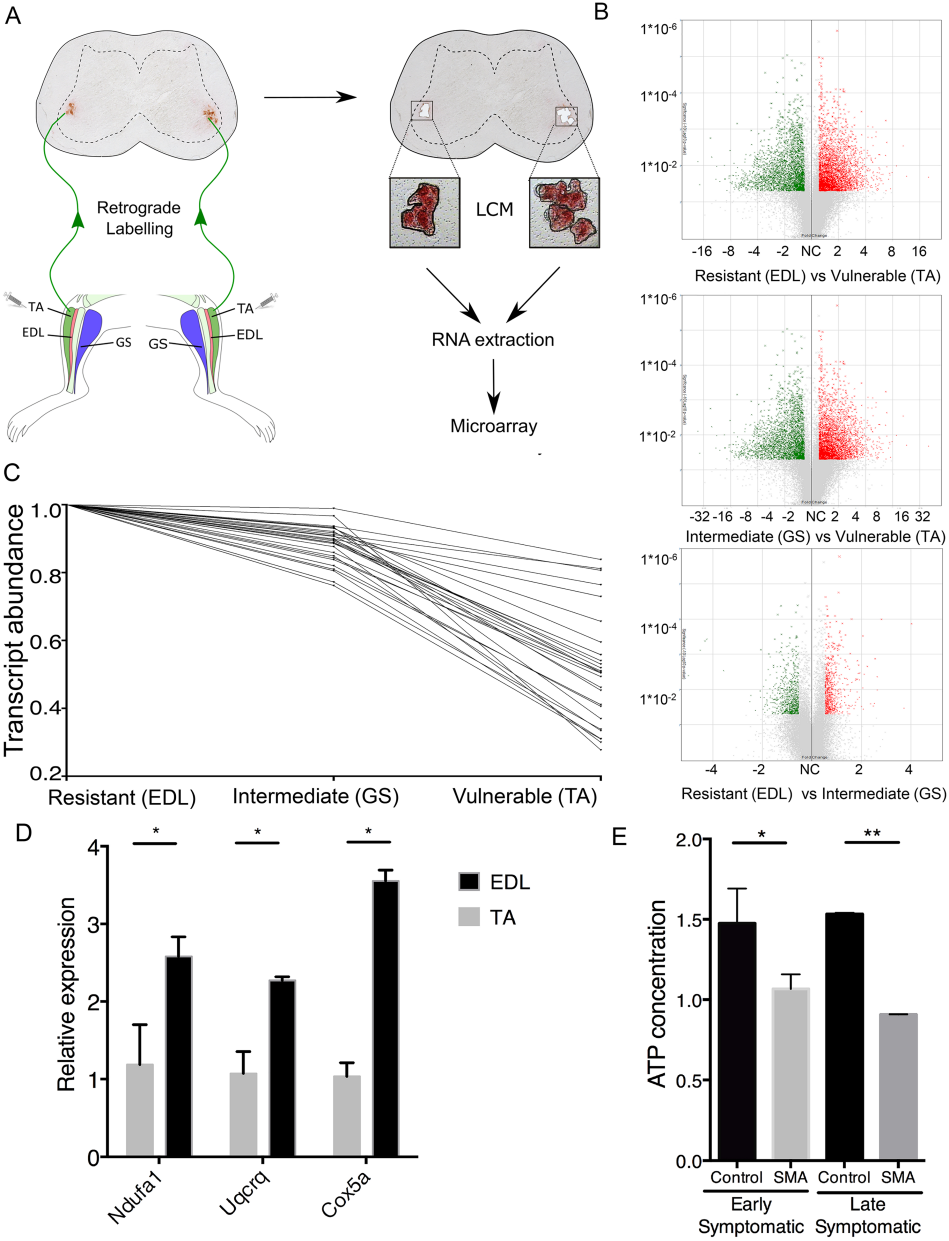
Microarray analysis of differentially vulnerable motor neuron pools reveals fundamental differences in their basal molecular composition. (A) Schematic illustration of experimental design (TA, tibialis anterior; EDL, extensor digitorum longus; GS, gastrocnemius; Vul, vulnerable MNs, Res, resistant MNs; Int, intermediate phenotype MNs). (B) Volcano plots of differentially expressed transcripts in resistant compared to vulnerable MN pools, intermediate compared to vulnerable MN pools, and resistant compared to intermediate MN pools. (C) Ratio trending analysis: transcripts that were significantly changed (p<0.05) between resistant (EDL) and vulnerable (TA) groups with a differential trending value in the intermediate (GS) group were first identified after which the data set underwent enrichment analysis to reveal enriched biological pathways. Graph shows an example of genes in one enriched biological pathway (mitochondrial electron transport chain genes). Note that transcripts showed highest expression levels in resistant (EDL) neurons, with a decreasing level of expression as the vulnerability status of the groups increased (GS through to TA). (D) qPCR validation for 3 distinct mitochondrial genes confirming up-regulation in disease-resistant MN pools (N = 3), Unpaired two tailed student *t-test* (* P<0.05). (E) Bar chart (mean & s.e.m.) showing a reduction in ATP in the spinal cord of early and late-symptomatic SMA mice compared to littermate controls using an ATP assay (N = 3 spinal cords per genotype).

In order to perform comparative gene expression profiling on pure populations of RNA from isolated MN pools with known differential susceptibility to SMA, we modified an established retrograde labelling approach to allow selective isolation of MN pools from the spinal cord. Wheat Germ Agglutinin (WGA) was injected into anatomically distinct muscle groups, resulting in labelling of the innervating MN cell bodies within the spinal cord [36] (Fig 1A). Given that mRNA is transcribed in the cell body and then subsequently transported either in its mRNA form or as a translated protein, we reasoned that differences in relative transcript abundance identified in MN cell bodies would reflect molecular changes potentially impacting on all cellular compartments of the MN.

We labelled MN pools innervating three distinct muscle groups, all located in the hind-limb of the mouse, which we have previously shown to have differential vulnerability status in SMA using endplate occupancy as a readout for degeneration in SMA (S1 Fig) [28]. We identified MN pools projecting to the disease-resistant *extensor digitorum longus* (EDL) muscle, intermediate *gastrocnemius* (GS) muscle, and vulnerable *tibialis anterior* (TA) muscle [28]. This meant that, as far as possible, any potential confounding factors such as muscle position in the body, nerve length, and motor neuron soma location in lumbar spinal cord, were standardized between groups (Fig 1A).

Comparative gene expression profiling studies were performed in young (post-natal day 14), healthy wild-type mice so to ensure that we were detecting only basal differences in the molecular composition of MN pools, without any secondary changes due to aging or disease-related stimuli. Non-toxic WGA was micro-injected into one of the three muscles (EDL, GS or TA) in each mouse (both hindlegs), using a total of three mice and six muscle injections for each muscle type. Two days after injection, the spinal cord was dissected and RNA was isolated from retrogradely labelled pure populations of motor neuron cell bodies using laser capture microdissection (LCM) prior to transcriptional profiling [37] (Fig 1A).

Initial analyses confirmed significant differences in basal gene expression profiles between the three distinct MN pools (Fig 1B). In order to identify transcriptional changes directly correlating with the relative disease vulnerability status of a MN pool, a trending analysis was employed to identify transcripts that differed across all three groups according to their relative vulnerability. Transcripts differentially expressed between the most resistant group (EDL) and the most vulnerable group (TA) with a significance threshold of P<0.05 were initially identified. This primary filter revealed two sets of transcripts: 1) those that were deemed potentially protective, having higher expression in resistant EDL MN pools compared to vulnerable TA MN pools, and; 2) those that were deemed as potentially harmful, having higher expression in vulnerable MNs compared to resistant MNs. These genes were then subjected to a second filtering step in which transcript expression values were required to trend through the intermediate muscle group (GS), in the appropriate direction of vulnerability. For example, a transcript with increased expression in the resistant versus the vulnerable group had to additionally show an expression value in the intermediate (GS) group midway between the vulnerable (TA) and resistant group (EDL). For example, genes grouped by biological function, such as mitochondrial electron transport genes in Fig 1C, had to show a trending expression pattern corresponding the vulnerability of that MN pool. Any genes that did not trend through the intermediate GS muscle were removed from further analysis.

For subsequent analyses we focused on transcript changes that were higher in resistant (EDL) compared to the more vulnerable groups, in order to identify potential protective modifiers of the disease. This analysis identified 2,065 individual transcripts whose expression was higher in resistant (EDL) compared to the more vulnerable MN pools (GS and TA) (Microarray files available at http://www.ncbi.nlm.nih.gov/geo/; GSE86908). Thus, robust differences are present in the molecular composition of distinct MN pools under basal conditions, with the potential to directly impact on their response to degeneration-inducing stimuli (e.g. low levels of SMN protein) in SMA.

### Enrichment of mitochondrial/bioenergetic genes and pathways in disease-resistant motor neurons

To explore the molecular mechanisms through which differences in the basal transcriptional composition of MNs may influence their vulnerability in SMA, our candidate gene list was taken forward for bioinformatics analysis using Gene Ontology (http://geneontology.org/) and DAVID (https://david.ncifcrf.gov/) to identify genetic and biological pathway enrichment.

A significant enrichment of mitochondrial and bioenergetic pathway-related genes was identified in EDL (disease-resistant) MN pools compared to more vulnerable MN pools (Table 1). The primary function of mitochondria is to generate ATP from oxidative phosphorylation, efficient ATP generation is particularly critical for highly metabolic tissues including motor neurons that have high energy demand to support cellular functioning and importantly synaptic transmission [38–40]. Increased expression of several individual mitochondrial genes identified by the microarray analyses were therefore independently validated with quantitative PCR, confirming higher expression in disease-resistant MNs (Fig 1D). These findings were of particular interest in the context of SMA as recent studies have suggested that mitochondrial abnormalities in both MNs and skeletal muscle may represent a pathological feature of SMA [41–45]. Moreover, spinal MNs from SMA patient induced pluripotent stem cells (iPSCs) revealed reduced mitochondrial number, area and transport [46]. Our analyses revealed an enrichment of mitochondrial genes particularly associated with oxidative phosphorylation, cellular respiration and generation of metabolites and energy in the resistant motor neuron pools, identifying a potential role for ATP-dependent pathways in regulating selective vulnerability of MNs in SMA (Table 1).

**Table 1:** Enrichment analysis of microarray data reveals biological pathways changed between disease-resistant and vulnerable motor neuron pools.

Gene Ontology Term	P-Value (p<0.05)	False discovery rate (FDR)
GO:0006091~generation of precursor metabolites and energy	4.47E-30	7.81E-27
GO:0022900~electron transport chain	2.61E-22	4.55E-19
GO:0045333~cellular respiration	1.83E-07	3.20E-04
GO:0005739~mitochondrion	1.74E-41	2.48E-38
GO:0019866~organelle inner membrane	6.41E-35	9.12E-32
GO:0005743~mitochondrial inner membrane	1.11E-33	1.58E-30
GO:0031966~mitochondrial membrane	1.38E-29	1.96E-26
GO:0005740~mitochondrial envelope	1.71E-29	2.43E-26
GO:0044429~mitochondrial part	5.89E-27	8.38E-24
GO:0070469~respiratory chain	6.60E-27	9.39E-24

Enrichment analysis of transcripts differentially expressed between differentially vulnerable motor neuron pools identified higher expression of mitochondrial and ATP producing genes in more resistant (EDL) pools compared to the intermediate (GS) and vulnerable (TA) groups using DAVID Bioinformatics resources 6.7 software (https://david.ncifcrf.gov/).

Given the association identified between the basal expression levels of ATP-dependent pathways and the vulnerability of MN pools to SMA, we wanted to confirm that similar ATP-dependent pathways were activated in response to disease-triggers in SMA. ATP assays were therefore performed on spinal cords extracted from early and late-symptomatic SMA mice and littermate controls. Levels of ATP were significantly reduced in SMA spinal cords compared to control both in early and late symptomatic mice suggesting a reduction in ATP production capabilities in SMA (Fig 1E). Taken together, these data show that expression levels of genes involved in mitochondrial/bioenergetics pathways are enriched in disease-resistant MN pools under basal conditions, with ATP-dependent pathways being adversely affected during the pathogenesis of SMA. This is particularly interesting given a recent report where SMA mouse motor neurons were found to have key differences in the expression of mitochondrial bioenergetic genes compared to controls, suggesting a wider role of bioenergetics in the pathogenesis of SMA [45].

### Mitochondrial abnormalities are present in a zebrafish model of SMA

To investigate the potential influence of ATP-dependent bioenergetics on MN pathology in SMA *in vivo*, we used an established *smn* knockdown zebrafish model of SMA using an antisense translation blocking morpholino [47–49]. These *smn* morphant zebrafish embryos have a robust MN phenotype (visualized in the Tg(*hb9*:GFP) line), where caudal primary motor axons fail to grow out in a defined straight direction into the developing somites and instead have axons with excessive branching and/or truncation. In severe cases, the motor axon fails to grow altogether. Reducing Smn protein levels using morpholino, synthetic microRNAs or a stable mutant line results in the same motor axon outgrowth phenotype identifying specificity and reproducibility of the phenotype [47, 50, 51].

**Fig 2:**
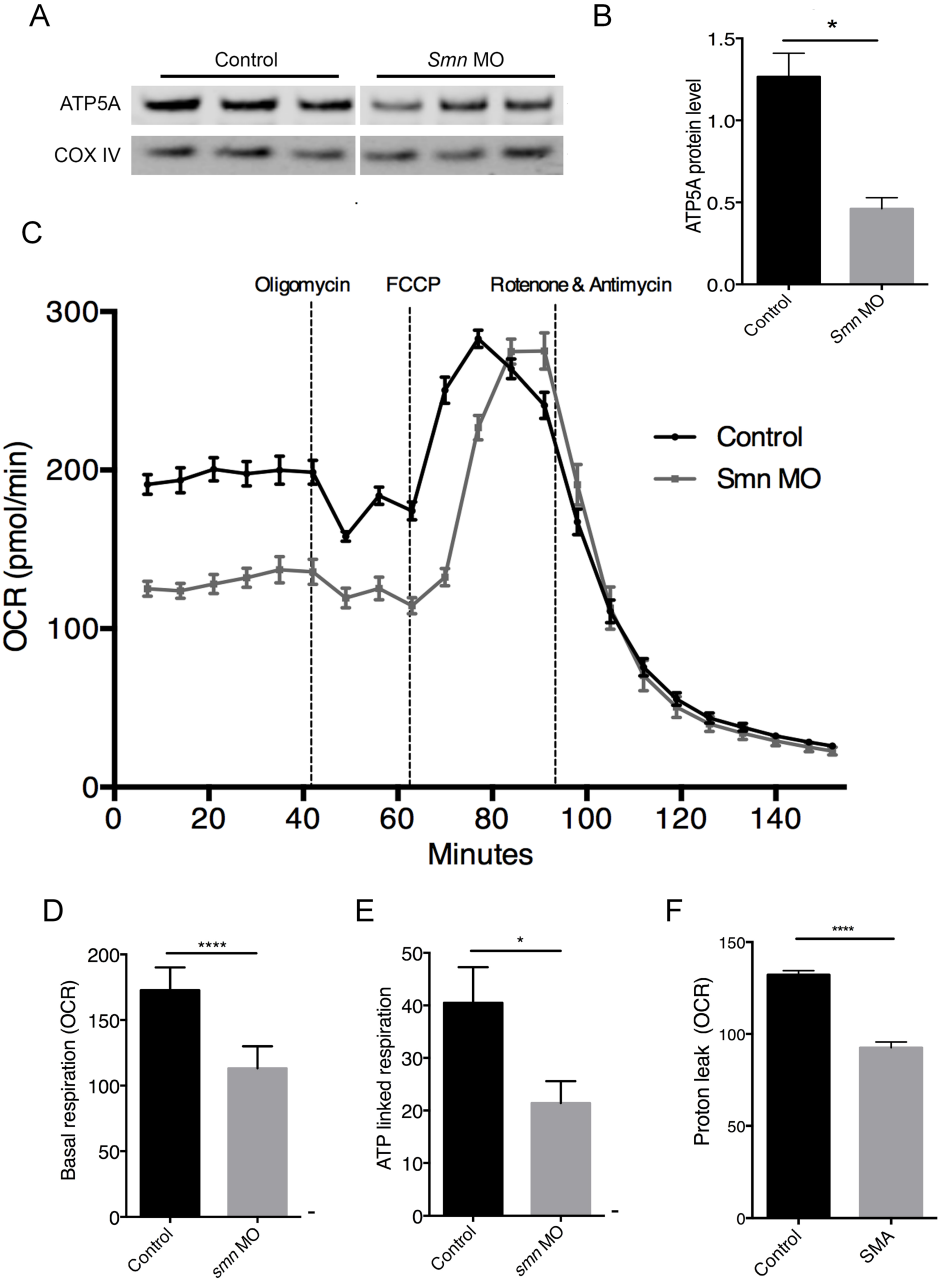
Mitochondrial dysfunction occurs in smn morphant zebrafish. (A) Levels of ATP5A protein, a subunit of mitochondrial membrane ATP synthase, were significantly reduced in *smn* morphant zebrafish. (B) Levels were quantified using fluorescent Western blotting and normalized to COXIV loading control (N = 3 per group, batches of 30 pooled zebrafish embryos per lane). (C) Mitochondrial oxygen consumption rates (OCR) of control and *smn* morphant 24 hpf zebrafish analyzed using the Seahorse XF24 analyser showed mitochondrial bioenergetic defects (D) Basal respiration was significantly reduced in *smn* morphants compared to controls. (E) ATP linked respiration was significantly reduced in *smn* morphants compared to controls. (F) Mitochondrial proton leak was also reduced in *smn* morphants compared to controls. N = 14 per group) Unpaired two-tailed student *t-test* * P<0.05, ** p<0.01 *** p<0.001.

Levels of ATP synthase subunit alpha (ATP5A), a subunit of mitochondrial membrane ATP synthase which produces ATP from ADP, was significantly reduced in *smn* morphant zebrafish embryos (Fig 2A and 2B). This suggests that mitochondrial ATP biogenesis defects were occurring in *smn* morphant zebrafish, similar to those previously identified in mice [45, 46]. Western blotting on pooled injected embryos confirmed the efficiency of the morpholino knockdown of the *smn* transcript compared to control embryos (S2 Fig). To investigate whether mitochondria showed signs of mitochondrial respiratory deficits in SMA zebrafish, a Seahorse XF analyzer was used to measure oxygen consumption rate (OCR), an indicator of oxidative phosphorylation and therefore mitochondrial respiration (Fig 2C). A significant reduction in basal respiration was observed in *smn* morphants at 24 hpf, where motor axon phenotypes are first visible, suggesting that the *smn* morphants were respiring at a significantly lower level to their age matched controls (Fig 2C and 2D). Addition of oligomycin, to inhibit the ATP synthase complex of the electron transport chain, can be used to calculate how much of the oxygen consumption is linked to ATP generation. Addition of oligomycin revealed that *smn* morphants have lower ATP linked respiration than control embryos (Fig 2C and 2E). Proton leak was also significantly reduced in *smn* morphants (Fig 2C and 2F). These data indicate that there is mitochondrial ATP generation abnormalities in situations of low Smn *in vivo*, supporting previous *in vitro* findings from mouse primary motor neurons [45].

### Increasing mitochondrial biogenesis rescues motor neuron phenotypes in a zebrafish model of SMA

**Fig 3:**
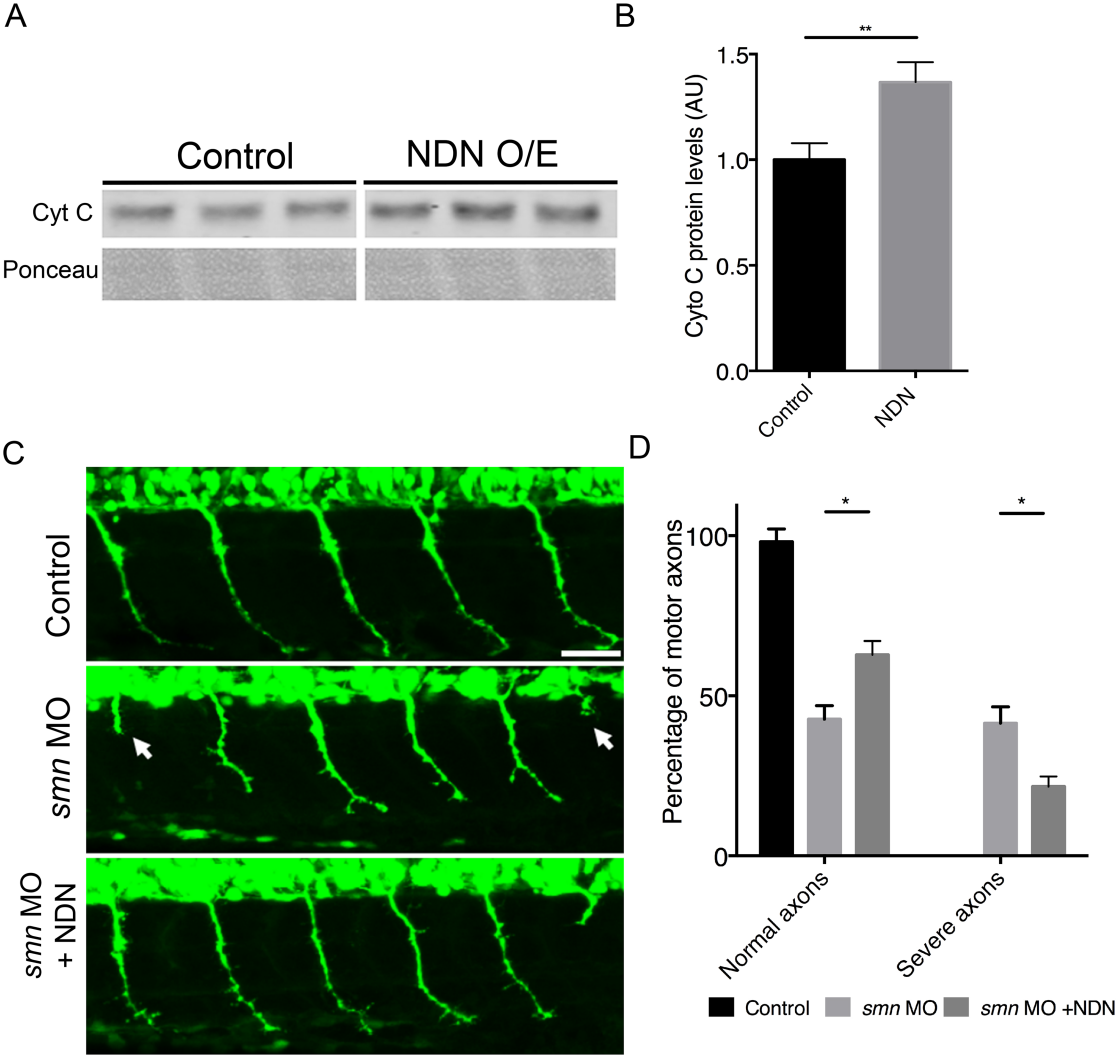
Overexpression of necdin ameliorates the motor axon outgrowth phenotype in *smn* morphant zebrafish. (A) Western blotting of cytochrome C, an electron transport chain protein showed an increase in NDN overexpressing embryos suggesting an increase in mitochondrial biogenesis. (B) Cyt C protein levels were quantified relative to a loading control. (C) Representative confocal micrographs of motor neuron axons exiting the spinal cord in control (top), *smn* morphant (middle) and *smn* morphant over-expressing Ndn (bottom) Tg(*hb9*:GFP) zebrafish embryos. Note the presence of the axonal outgrowth phenotype associated with *smn* knockdown (arrow heads) is reduced in the Ndn expressing animals. Scale bars = 50 μM. (D) Bar chart (mean & s.e.m.) showing a significant increase in the number of normal MNs, and a concomitant significant decrease in the number of severely affected MNs, in co-injected *smn* MO and Ndn mRNA embryos compared to single *smn* MO injected embryos at 30 hpf. Unpaired two-tailed student *t-tests*; * p<0.05, ** p<0.01 *** p<0.001. N = 20 embryos per experimental group.

Using the same model, we modulated ATP-dependent pathways by increasing mitochondrial biogenesis, thereby replicating the molecular profile of endogenously disease-resistant MNs. Necdin (NDN), a MAGE family protein expressed in all neuronal cells [52], has recently been demonstrated to promote neuronal mitochondrial biogenesis [53]. Furthermore, its overexpression was previously found to have a neuroprotective effect in dopaminergic neurons exposed to complex I inhibitors [53]. Overexpression of NDN produced an increase in cytochrome C expression, demonstrating an increase in mitochondrial biogenesis (Fig 3A and 3B) [54]. We found that enhancing mitochondrial biogenesis by overexpressing NDN in our *smn* morphant zebrafish embryos resulted in a significant rescue of axonal outgrowth phenotypes (Fig 3C). There was a significant increase in the numbers of MNs with normal axonal outgrowth (Fig 3D) and a concomitant significant decrease in the number of motor neurons with missing or truncated axons (severe axons) (Fig 3D). Injection of a control morpholino did not result in any axonal outgrowth phenotypes confirming phenotype specificity upon *smn* knockdown (S3 Fig). Thus, enhancement of mitochondrial biogenesis (replicating the molecular profile of disease-resistant motor neurons) was sufficient to ameliorate axonal outgrowth phenotypes in *smn* morphant zebrafish.

### Bioenergetic-dependent regulation of motor neuron vulnerability is mediated by PGK1

Given the identification of bioenergetic pathway gene enrichment in disease-resistant MNs, and the rescue of motor axon phenotypes *in vivo* when mitochondrial biogenesis was increased, we wanted to further explore the mechanisms through which other bioenergetic pathways are mediating MN vulnerability. We hypothesized that those MNs capable of better meeting increased energy demands, especially at the NMJ, were more likely to remain stable during disease progression, for example during energy stress induced by the presence of hypoxia in SMA [55].

**Fig 4:**
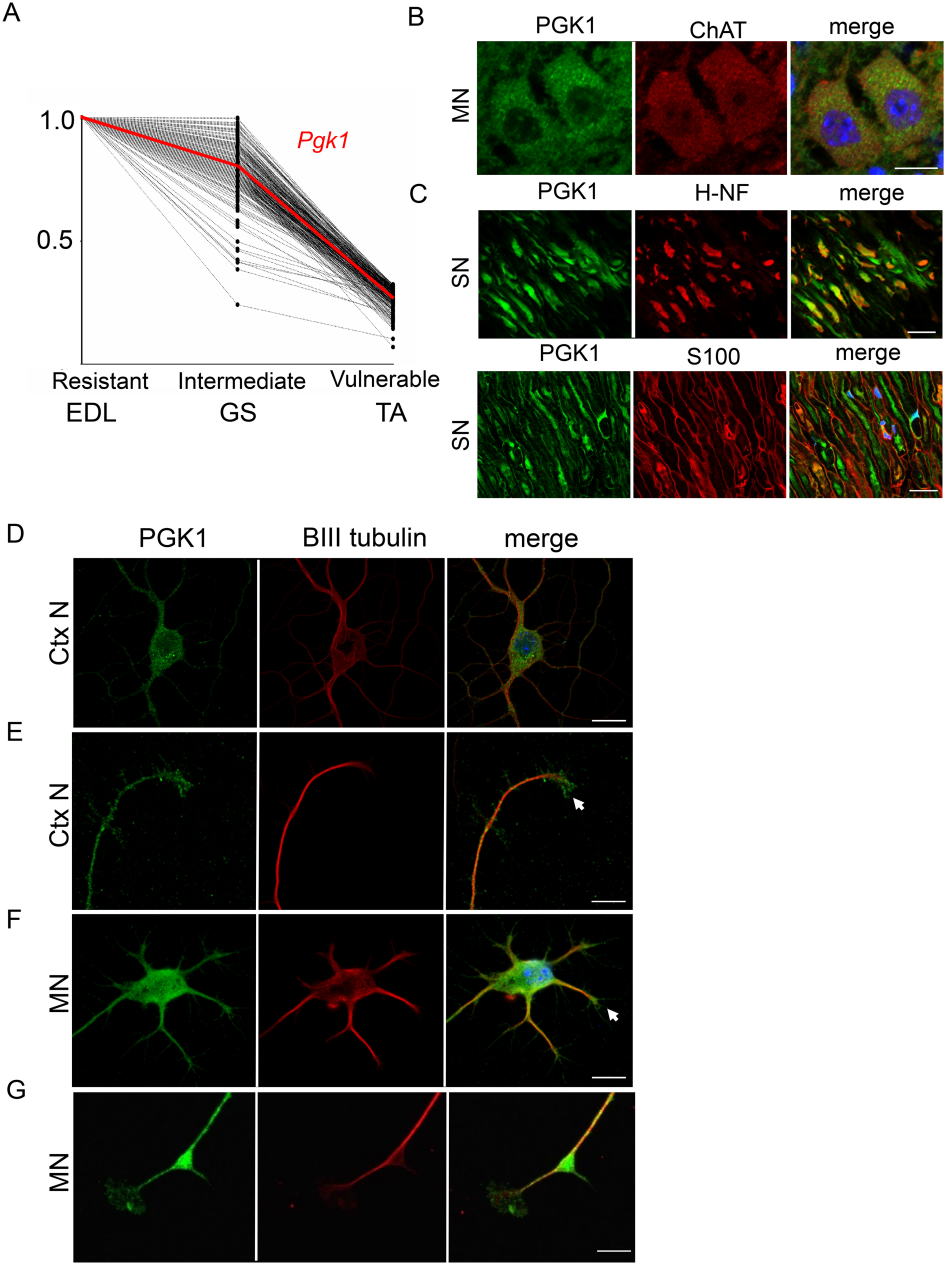
PGK1 is enriched in disease-resistant motor neuron pools, expressed in neuronal cells cellular and axonal compartments *in vivo* and *in vitro*. (A) Gene expression profile graph showing transcripts trending across differentially vulnerable motor neuron pools, with PGK1 highlighted. Note that *Pgk1* was 5-fold higher expressed in the EDL disease-resistant motor neuron pool compared to the TA vulnerable motor neuron pool, with expression levels trending through the GS intermediate pool. (B) Representative confocal micrographs showing expression of PGK1 in the cytoplasm of motor neurons (MN) in mouse spinal cord. Scale bars = 20 μM. (C) Expression of PGK1 was also detected in the majority of axons in the sciatic nerve (SN), being localised alongside neurofilament (H-NF; upper panels) but not co-localising with glial S100 label (lower panels). Scale bars = 5 μM (D) *In vitro* analysis showed expression of PGK1 in the cell body and axonal processes of mouse cortical neurons (CtxN). Scale bars = 30 μM. (E) Expression of pgk1 was detected in the axonal nerve terminals of mouse cortical neurons (CtxN). Scale bars = 15 μM. (F) Expression of PGK1 was also found in mouse primary motor neuron (MN) cell bodies and axonal compartments (arrow). Scale bars = 30 μM. (G) Expression of Pgk1 in the axonal terminals/growth cones (arrow) of mouse primary motor neurons (MN). Scale bars = 15 μM.

Phosphoglycerate kinase 1 (*PGK1*) was one particular gene of interest in our microarray dataset, identified as having increased expression in resistant motor neurons (Fig 4A). PGK1 is a key glycolytic enzyme that catalyzes the conversion of 1,3-diphosphoglycerate to 3-phosphoglycerate, generating the first molecule of ATP in the glycolytic process. Mutations in *PGK1* have been associated with a range of phenotypes in human patients, including severe Central Nervous System (CNS) defects and muscle fatigue [56, 57]. Furthermore, glycolysis has been shown to be critical for synaptic function [58], generating local ATP for vesicular recycling and therefore synaptic transmission, a process which is defective in SMA [22]. Glycolysis at the NMJ is also required during energy stress, including mitochondrial dysfunction, to meet acute energy demands at the synapse [58].

Expression of PGK1 in motor neuron cell bodies was confirmed using immunohistochemistry on spinal cord sections (Fig 4B). Expression of PGK1 was also found in peripheral axons in the sciatic nerve, where it colocalised with neurofilament proteins but not the glial cell marker S100 (Fig 4C), suggesting that PGK1 is present throughout the extensive cytoplasm of MNs *in vivo*. To confirm additional distal localization of PGK1, immunohistochemistry was carried out in both cultured mouse cortical neurons and primary motor neurons, confirming robust expression throughout the axon (Fig 4D and 4F) and at neuronal terminals (Fig 4E and 4G arrows) in both cell types. Co-staining with GAPDH in primary MNs also revealed staining in both axons and growth cones, suggesting that PGK1 expression in distal compartments is due to its glycolytic roles in generating local ATP (S5 Fig).

**Fig 5:**
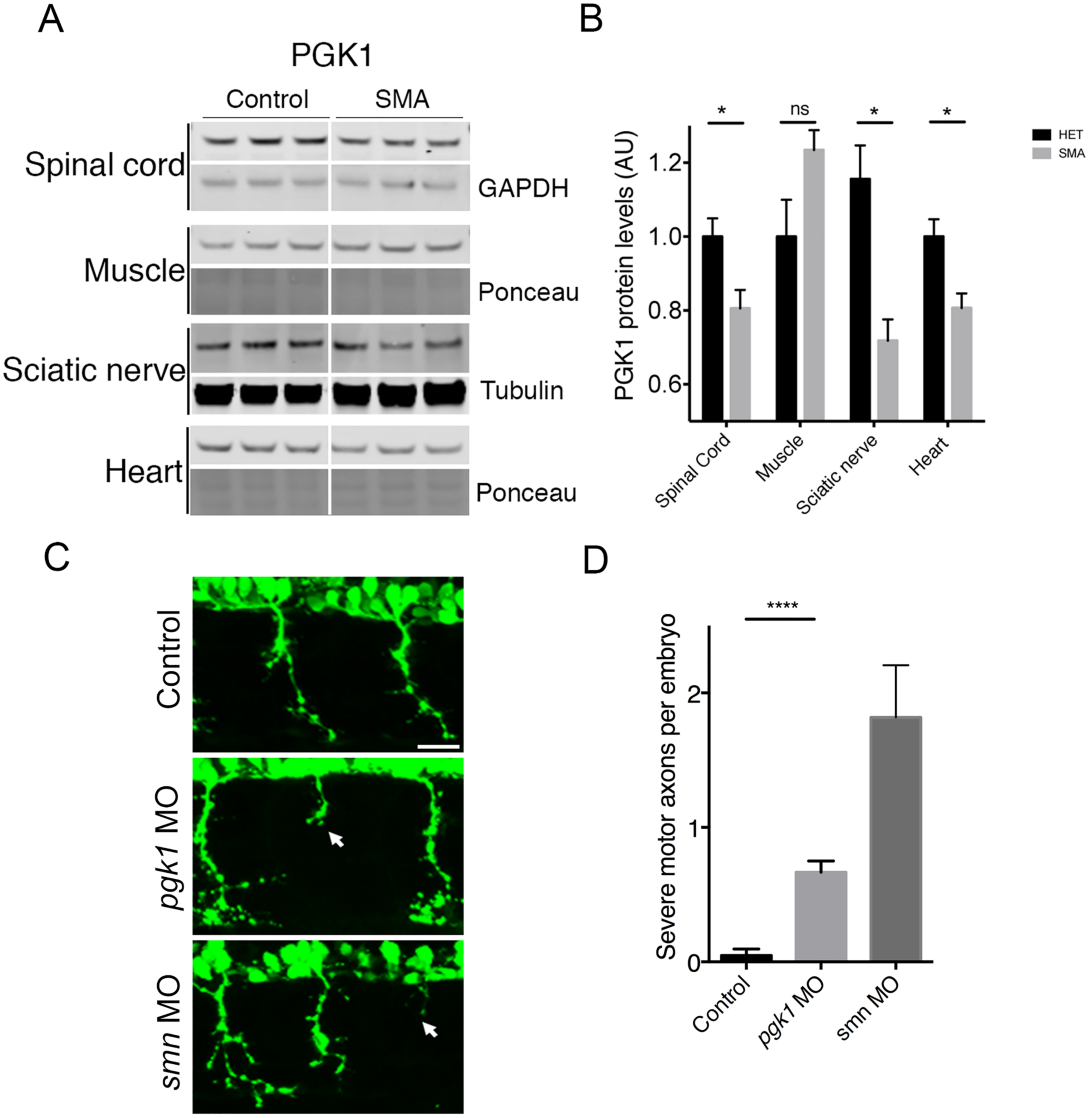
Pgk1 expression is pathologically relevant in mouse and zebrafish models of SMA. (A) Expression of PGK1 protein in the spinal cord, skeletal muscle, sciatic nerve and heart of late-symptomatic P8 SMA mice. Protein levels were quantified and normalized to an appropriate loading control. (B) Bar chart (mean & s.e.m.) showing a significant reduction in PGK1 protein levels in SMA mouse spinal cord and sciatic nerve. N = 6 SPC per genotype. N = 3 muscle per genotype. N = 7 sciatic nerves per genotype. N = 3 hearts per genotype (C) Knockdown of Pgk1 in zebrafish induced an axonal outgrowth phenotype (middle panel arrow) similar to smn knockdown (arrow bottom panel) and also produced swellings in the tips of outgrowing axons indicative of axonal transport deficiencies. Scale bars = 50 μM (D) Quantification of axonal outgrowths showed a significant increase in truncated motor axons in pgk1 and smn morphants compared to controls. (E) Efficiency of *pgk1* knockdown in embryos was shown by western blot embryos normalized to an appropriate loading control (N = 3 per group, batches of 30 pooled zebrafish embryos per lane). N = 20 embryos per group. Unpaired two-tailed students *t-test* * p<0.05, ** p<0.01 *** p<0.001 **** p<0.0001.

To further explore the potential contribution of PGK1 to SMA disease pathogenesis, we next examined PGK1 levels in different tissues from late-symptomatic SMA mice (Fig 5A). PGK1 levels were significantly reduced in SMA mouse spinal cord and sciatic nerve (Fig 5B). In contrast, PGK1 levels were not significantly changed in skeletal muscle (Fig 5B). We also observed a reduction of PGK1 in the heart of SMA mice, another highly metabolic tissue and affected tissue in severe SMA, suggesting that other tissues with high energy requirements may be affected. This identifies a potential role for energy demand in modulating disease response and vulnerability across a range of tissues (Fig 5A and 5B). Similarly, a significantly reduced level of PGK1 in the spinal cord was also observed at an early-symptomatic time point in SMA mice compared to controls (S6 Fig).

Next, to establish any potential role for PGK1 in modulating motor neuron stability, we examined the consequence of reducing PGK1 levels on healthy MNs *in vivo*. Knockdown of *pgk1* in wild-type zebrafish embryos using a morpholino was sufficient to induce an SMA-like axonal outgrowth phenotype, characterized by greater numbers of truncated motor axons and abnormal axon branching in *pgk1* morphant embryos compared to controls (Fig 5C and 5D). Efficiency of Pgk1 knockdown in zebrafish embryos was determined by western blot, confirming that protein levels were reduced by 50% (Fig 5E). The concentration of injected pgk1 morpholino resulted in a dose dependent increase in severe motor axons suggesting specificity of the phenotype (S7 Fig). Thus, experimental suppression of Pgk1 levels was sufficient to destabilise MNs *in vivo*, consistent with higher basal levels of Pgk1 conferring protection and stability in disease-resistant MNs.

**Fig 6:**
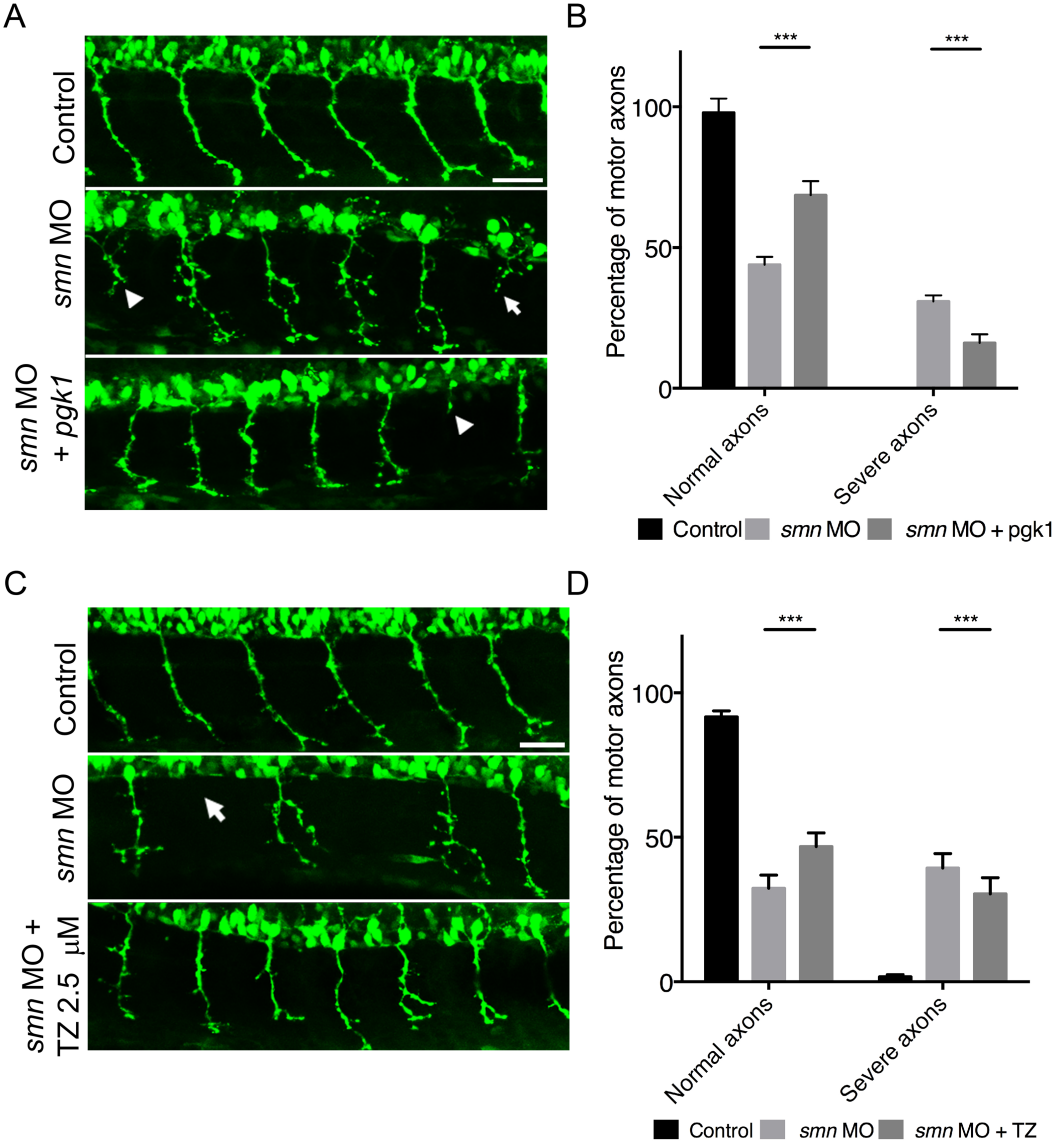
Overexpression or pharmacological activation of pgk1 rescues motor neuron phenotypes in smn morphant zebrafish. (A) Representative confocal micrographs of primary motor neuron axons exiting the spinal cord in control (top), *smn* morphant (middle) and *smn* morphant over-expressing *pgk1* (bottom) Tg(*hb9*:GFP) zebrafish embryos. Note the presence of an axonal outgrowth/branching phenotype associated with *smn* knockdown (arrow heads) that is reduced in the *pgk1* over-expressing animals. Scale bars = 50 μM. (B) Overexpression of Pgk1 in *smn* morphant zebrafish at 30 hpf led to a significant increase in normal motor axons and significant decrease in severe axonal outgrowth phenotypes compared to single *smn* MO injected embryos. (C) Representative confocal micrographs of motor neuron axons exiting the spinal cord in control (top), *smn* morphant (middle) and *smn* morphant treated with 2.5 μM terazosin (bottom) Tg(*hb9*:GFP) zebrafish embryos. Note how the presence of the axonal outgrowth/branching phenotype associated with *smn* knockdown (arrow heads) is reduced in the terazosin-treated animals. (D) Bar chart (mean & s.e.m) showing activation of Pgk1 by treatment with 2.5 μM terazosin in *smn* morphant zebrafish at 30 hpf led to a significant increase in normal motor axons and significant decrease in severe axonal outgrowth phenotypes compared to untreated *smn* MO injected embryos. Unpaired two-tailed student *t-tests* * p<0.05, ** p<0.01 *** p<0.001. n = 20 embryos per group.

Finally, we wanted to establish whether therapeutic targeting of Pgk1 would be sufficient to confer a disease-resistant phenotype on MNs in *smn* morphant zebrafish. We initially used a genetic over-expression approach to increase Pgk1 levels in *smn* knockdown zebrafish. Overexpression of p*gk1* mRNA in *smn* morphant zebrafish resulted in a significant rescue of the axonal outgrowth phenotype (Fig 6A arrows), with fewer MN axons displaying a truncated outgrowth phenotype and greater numbers of normal MNs (Fig 6B).

To confirm these findings and establish whether this pathway is amenable to more therapeutically-relevant (pharmacological) targeting strategies, we repeated our SMA zebrafish experiments using an FDA-approved small molecule, terazosin (TZ). TZ binds Pgk1 and activates its enzymatic activity [59], with previous studies revealing a potential for conferring neuroprotection in animal models of sepsis and stroke [59]. Activation of Pgk1 using 2.5 μM of TZ significantly rescued the MN axonal outgrowth phenotypes in *smn* morphant zebrafish (Fig 6C and 6D). No morphological or developmental defects were observed with TZ treatments, suggesting no off-target effects (S8 Fig). Thus, targeting of Pgk1, either genetically or using a small molecule approach, can protect motor neurons from SMA-induced pathology *in vivo*. The similar findings obtained between our genetic and pharmacological experiments also suggest that the effects of terazosin were resulting directly from targeting pgk1, rather than additional, off-target, effects.

## Discussion

Differential vulnerability of MN pools within an individual is a significant feature of the pathogenesis of SMA. Here, we demonstrate that differences in the basal molecular composition of MN pools correlate directly with their relative vulnerability or resistance during disease. Gene expression profiling of three anatomically-distinct MN pools with differing vulnerability in SMA revealed that disease-resistant MNs had higher basal expression of genes associated with bioenergetics pathways, which were subsequently found to be perturbed in SMA. Targeting of these identified bioenergetic pathways, initially by enhancing global mitochondrial biogenesis, was found to rescue MN defects in *smn* morphant zebrafish. Similarly, focused targeting of non-mitochondrial bioenergetic pathways, by increasing the levels or activity of phosphoglycerate kinase 1 (Pgk1), was found to rescue MN phenotypes associated with *smn* knockdown.

Several studies investigating the underlying factors influencing selective vulnerability of MNs have been conducted, highlighting it as a keen area of interest in the field of motor neuron disease research. Taken together with our current findings, these suggest that the discovery of inherent protective modifiers could be fundamental to the development of future successful combinatorial therapies [33, 60–63]. It is of particular interest to note that mitochondrial pathways have been previously highlighted as a potential disease modifier in ALS [64]. Taken together with our current findings from SMA, this suggests that differences in basal bioenergetic function could be a fundamental and conserved determinant of MN vulnerability across a range of different neurodegenerative conditions. Indeed, mitochondrial dysfunction has been highlighted as an important feature across a broad spectrum of neurodegenerative disorders [40, 65], unsurprising given their extensive roles in cellular homeostasis [38], critically ATP generation, particularly in neurons [66]. Moreover, it has recently been reported that treating an ALS mutant mouse model with Triheptanoin to aid ATP generation through improving oxidative phosphorylation protected motor neurons [67], again suggesting that therapeutic targeting of ATP production could be beneficial.

**Fig 7:**
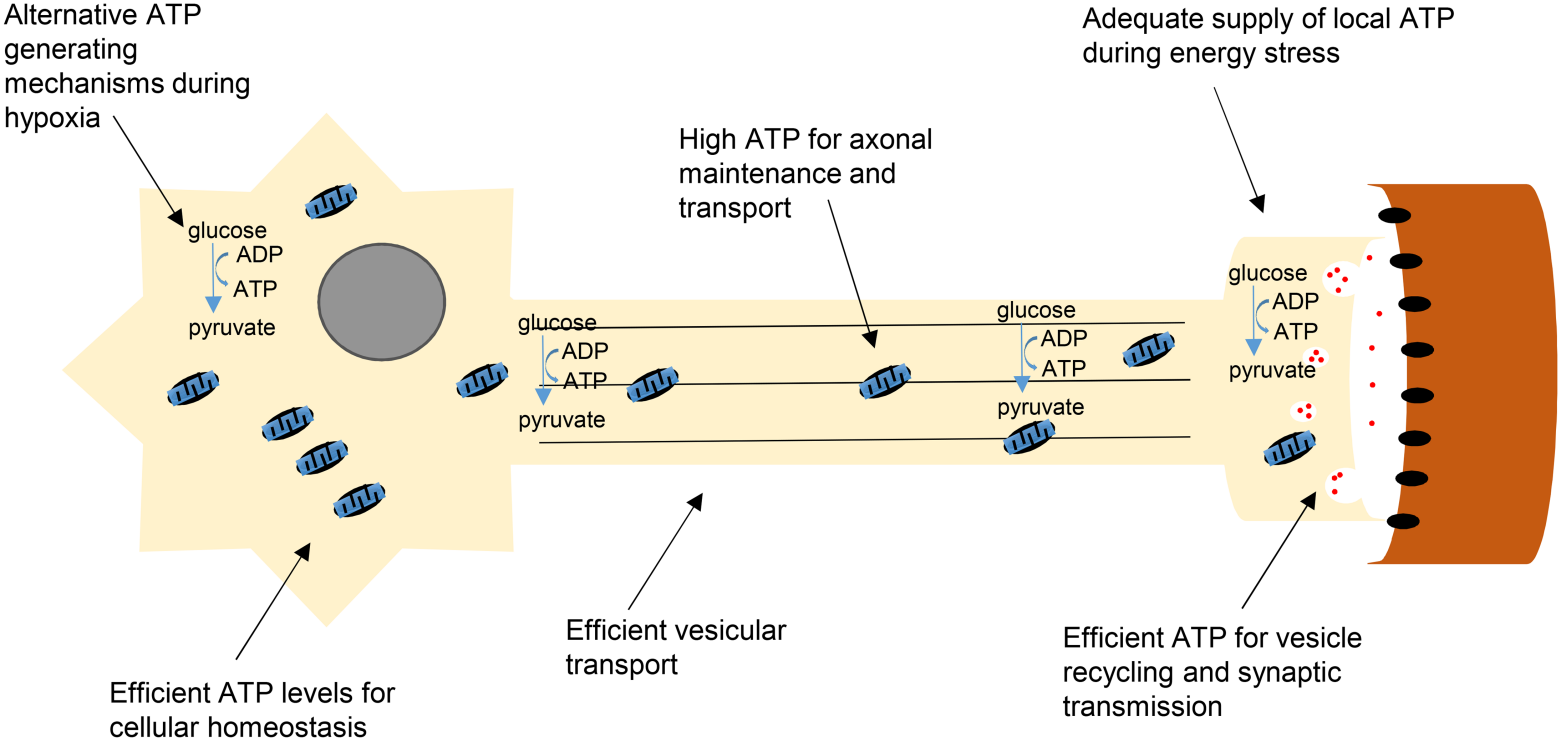
Summary model showing ATP-generating pathways likely to influence the vulnerability status of MNs in SMA. Efficient ATP generation through different processes maintains levels in energy demanding MNs, critical for their function and survival during disease. Certain motor neuron pools possess inherently higher bioenergetic capacities that provide protection during cellular insult both at the cell body and at the NMJ. Efficient ATP generated from mitochondria allows cellular homeostasis to be maintained. During hypoxia, glycolytic pathways are employed to meet acute ATP demand, particularly at the NMJ, critical for vesicular recycling and synaptic transmission. Mitochondrial transport along the axon provides local ATP to maintain axonal integrity. Glycolytic machinery present along the axon allows for fast vesicular transport down to the NMJ to deliver packaged proteins for pre-synaptic function.

From the SMA perspective, our findings add significant additional weight to a growing body of evidence suggesting that mitochondria are likely to be a critical mediator of disease pathogenesis. For example, a recent study using RNA sequencing to compare healthy and SMA MNs *in vitro* identified differential expression levels of several mitochondrial and bioenergetic genes. Furthermore, they confirmed that in SMA MNs, mitochondrial ATP respiration and mitochondrial transport were both reduced [45]. We show similarly here *in vivo* that *smn* morphant zebrafish have reduced ATP linked mitochondrial respiration (Fig 2). Taken together with our finding that targeting of bioenergetic pathways rescues MN defects in *smn* morphant zebrafish, this suggests that MNs with a greater bioenergetic capacity have higher disease resistance due to greater energy supply for critical MN functions (Fig 7).

During hypoxia or mitochondrial dysfunction, cells become more reliant on glycolysis to produce ATP to meet acute energy demands. Both mitochondria and glycolysis are responsible for generating the majority of cellular ATP. Mitochondria and glycolytic machinery are both present at neuronal synapses providing energy for synaptic transmission and vesicular recycling [68], with enrichment of glycolytic machinery found at neuronal terminals providing local ATP when under high energy demands or in smaller synapses where mitochondria are absent [58]. As synapses are known to be particularly susceptible in SMA, it is interesting to note that we have previously found glycolytic proteins to be altered in synapses from SMA mouse brain [13]. Moreover, given that defects in vasculature lead to hypoxic conditions (causing reduced oxygen availability for mitochondrial oxidative phosphorylation) throughout the neuromuscular system in SMA [55, 69], it is likely that those cells more capable of meeting energy demands by alternative methods may be more resistant to the disease.

This contribution of glycolytic pathways to neuronal and synaptic health is consistent with our finding that *Pgk1*, a key glycolytic gene, showed a 5-fold enrichment in disease-resistant MNs (EDL). Mutations in *PGK1* present with a wide range of clinical manifestations in human patients, including CNS defects and muscle defects (both tissues with high metabolic demands that are also affected in SMA) [56, 57]. We confirmed robust expression of PGK1 in the cytoplasm of MNs and at axon terminals, suggesting that it is an important contributor to bioenergetic pathways in this cell type (Fig 4). Moreover, reducing levels of *pgk1* in wild-type zebrafish resulted in an *smn* morphant axonal outgrowth phenotype, confirming a direct influence on the regulation of MN stability and health (Fig 5C and 5D).

Previous studies using FDA approved terazosin (TZ), which activates PGK1, revealed protective effects in both stroke and sepsis models, most likely due to enhanced stress resistance [70], in keeping with our findings from SMA models. Moreover, other studies have suggested that its protective function may be additionally mediated by effects on the glycolytic machinery and its regulation of fast synaptic vesicular transport along axons, required to provide local ATP at synaptic terminals [68, 70]. Whether PGK1 is protective due to increased vesicle transport in disease resistant motor neurons remains speculative, but it could be highly relevant in SMA given that axonal transport and ATP related deficiencies have been actively implicated in disease pathogenesis [11, 45, 71–73].

Importantly, our finding that elevation of Pgk1 levels or activity, via injection of *Pgk1* mRNA or treatment with terazosin respectively, robustly ameliorated MN pathology in *smn* morphant zebrafish provides an initial demonstration that these pathways are amenable to therapeutic intervention. Moreover, the fact that inherently ‘disease-resistant’ MNs already have higher levels of PGK1 would suggest that elevating levels across all neurons (albeit whilst remaining within physiological levels) would be a safe therapeutic intervention. Encouragingly, our terazosin experiments also suggest that there are existing FDA-approved drugs that can activate PGK1 enzymatic activity that may be suitable for repurposing for conditions such as SMA. As SMN-targeted therapies progress through clinical trials, showing promise to modify disease but likely falling considerably short of offering a ‘cure’ for SMA [74], it is becoming clear that additional, complementary therapies are going to be required [17]. Our findings suggest that targeting the bioenergetic status of MNs represent one attractive approach to develop such a combinatorial therapy. Finally, given that other highly metabolic tissues are affected in SMA patients and animal models, bioenergetic therapies could also be beneficial for non-MN pathology associated with the disease [25].

## Materials and methods

### Animals

Wild-type FVB mice (both male and female) at P14 were used for microarray experiments. The Taiwanese mouse model of severe SMA (*Smn*^*-/-*^*; SMN2*^*tg/o*^) on a congenic FVB background was used for SMA experiments [75], employing a breeding strategy previously described [76]. Age matched, phenotypically normal littermate (*Smn*^*+/-*^*; SMN2*^*tg/o*^) mice were used as controls, with all animals being retrospectively genotyped using standard PCR protocols [76]. Mouse breeding stocks were originally obtained from Jackson laboratories and were maintained in animal care facilities at the University of Edinburgh under standard specific pathogen free conditions. Zebrafish embryos were maintained using standard protocols at 28.5°C and staged by hours post-fertilization (hpf) [77]. Adult wildtype and Tg(hb9:GFP) zebrafish [78] were maintained in a fish facility at the University of Edinburgh according to standard methods.

### Synaptic vulnerability analysis and quantification

Muscles were dissected, processed and imaged as previously described [28]. In short at least 80 endplates per muscle per mouse were assessed in each muscle preparation in wildtype and SMA mice. For endplate occupancy counts, the occupancy of NMJs was determined by classifying endplates as either fully occupied (neurofilament and SV2 entirely overlie the endplate), partially occupied (neurofilament and SV2 cover less than 50% of the endplate), or vacant (no neurofilament or SV2 overlying the endplate).

### Retrograde labelling and tissue preparation for LCM

Nine FVB mice from the same wild-type FVB litter at p14 were divided into three groups according to the muscle location being injected, resulting in groups of 3 mice per muscle. Mice were anaesthetized and a small surgical incision was made in the skin of the hindleg. 20μg/ml of WGA-HRP (Vector Laboratories; PL-1026)) was injected into individual *tibialis anterior* (TA, vulnerable; 3μl), *gastrocnemius* (GS, intermediate; 3μl), and *extensor digitorum longus* (EDL, resistant; 1μl) muscles. Mice were sacrificed and perfused with PBS 48 hours after surgery. Spinal cords were immediately collected, embedded in optimal cutting temperature compound (OCT) and stored at -80°C. In order to minimise contamination from other cell types when performing laser capture microdissection, frozen sections (35 μm) were mounted onto PEN-membrane slides (Applied Biosystems, Foster City, CA). Tissue sections were fixed in cold acetone and then dehydrated using a cold graded ethanol series (75%, 95% and 100% for 30 seconds), and xylene for 3 min. Dehydrated sections were developed with NovaRED Peroxidase (HRP) Substrate (Vector Laboratories, Burlingame, CA) for 3 min. Those sections identified positively for retrograde labelling were then transferred to the Arcturus Veritas laser capture microdissection system (Applied Biosystems) for isolation of labelled MN soma.

### RNA isolation, amplification and microarray hybridization

Total RNA was extracted from isolated MNs using PicoPure RNA Extraction kit according to the manufacturer’s instructions (Applied Biosystems). Approximately 100 ng of total RNA was obtained from labelled MNs extracted from a single spinal cord using LCM. Before hybridisation, samples were subjected to linear amplification, and the products were analysed for quality (Agilent 2100 Bioanalyzer, RNA 6000 NanoChip) and quantity (NanoDrop 1000 Spectrophotometer) according to the GeneChip Pico Kit protocol (Affymetrix). In brief, the 100ng of total RNA was amplified with random primers containing a T7 polymerase promoter site. At the end of reactions, double-stranded cDNA was generated ready for *in vitro* transcription to synthesise copy RNA (cRNA). The cRNA (20 μg) was subsequently used as template for generating single-stranded cDNA (ss-cDNA), afterwhich 5.5μg of fragmented and biotin-labelled ss-cDNA was hybridised to one GeneChip Mouse Transcriptome Assay 1.0 (Affymetrix) for 16hr at 45°C, followed by stringent washing in a GeneChip Fluidics Station 400 (Affymetrix) and scanning using a GeneChip Scanner 30007G (Affymetrix). Datasets (.CEL files) taken forward for further analysis are freely available in the public database (http://www.ncbi.nlm.nih.gov/geo/; GSE86908).

### Microarray analysis

The.CEL files were processed using an Affymetrix Expression Console with the sst-RMA summarisation algorithm. To identify transcript changes correlating to susceptibility, statistically significant differences were determined by comparing three subtypes of differentially affected motor neuron pools (tri-wise or 3-way comparison) in Transcription Analysis Console 3.0 (Affymetrix). The variable gene list was then generated based on two cut-offs: 1) genes significantly different between EDL (resistant) and TA (vulnerable) groups, and 2) gene expressions fitting a trend either increasingly or decreasingly through EDL (resistant)-GS (intermediate)-TA (vulnerable). Gene Ontology (http://geneontology.org/) and DAVID (https://david.ncifcrf.gov/) were used to identify genetic and biological pathway enrichment in the data set.

### Quantitative RT-PCR

A proportion of the RNA amplified from laser-captured EDL and TA motor neurons was used in quantitative PCR to verify expression levels of 3 distinct mitochondrial genes, Ndufa1, Uqcrq and Cox5a. cDNA was synthesised using qScript cDNA synthesis kit according to the manufacturer’s protocol (Quanta). qPCR was performed using 12.5 ng cDNA, 1X SensiFast SYBR (Bioline) and optimised primers (Table 2), to a total volume of 20 μl. After the initial denaturation at 95°C for 2 min, cDNA was amplified by 40 cycles of 95°C for 5 sec and 63°C for 25 sec, on a CFX96 Real-Time PCR system (Bio-Rad). Gene expression levels were normalised to β-actin expression and ΔΔCt calculation was applied to generate relative concentration. The statistical analysis was performed using a two-tailed unpaired *t* test.

**Table 2:** Primer sequences used in quantitative PCR.

Gene symbol	Primer sequences	Conc.	Efficiency
*Ndufa1*	F, 5’GAGTAACGGTGCGGAGATGT3’ R, 5’CGTTGGTGAATTTGTGGATG3’	100nM	100%
*Uqcrq*	F, 5’GTCTACCTGATCTACACATGGG3’ R, 5’CAGAGAAGGGTCTTTCAGAGG3’	250nM	100%
*Cox5a*	F, 5’GCCGCTGTCTGTTCCATTC3’ R, 5’GCATCAATGTCTGGCTTGTTGAA3’	100nM	94%

### ATP assay

Spinal cords from early-symptomatic p5 and p8 late-symptomatic SMA mice and control mice were dissected and pooled by genotype to produce a total of 20mg of tissue. Spinal cords were homogenised and deproteinized using Perchorlic acid (PCA). Samples were then measured for ATP concentrations using the Abcam ATP assay kit, following manufacturer’s instructions (ab83355, Abcam).

### Zebrafish protein preparation

Wildtype AB stain one cell stage embryos were injected with 6ng of MO and left to develop until 48 hpf. Embryos were dechorionated and deyolked in 1ml of deyolking buffer (1/2 Ginzburg Fish Ringer without Calcium: 55 mM NaCl, 1.8 mM KCL, 1.25 mM NaHCO3). Embryos were pooled into batches of 30 embryos, with three replicate batches per experimental group. Embryos were pelleted at 300g for 30 seconds and the supernatant was discarded. Embryos were washed twice with wash buffer (110mM NaCL, 3.5 mM KCl, 2.7 mM CaCl2, 10 mM Tris pH 8.5) and cells pelleted at 300g for 30 seconds. The supernatant was removed and cellular pellet was stored at -80°C until use [79].

### Quantitative fluorescent western blotting

All protein (zebrafish embryos and mouse tissue) was extracted in RIPA buffer (ThermoScientific) with 1% protease inhibitor cocktail (Sigma) and homogenised. Protein concentration was determined by BCA assay (ThermoScientific). SDS-page was performed using 20% pre-cast NuPage 4–12% BisTris gradient gels (Life Technologies) and transferred to PVDF membranes by the iBlot 7 minute semi-dry blotting system (Life Technologies). Following blocking, membranes were incubated overnight at 4°C using mouse anti-SMN (1:1,000, BD biosciences, 610153), rabbit anti-PGK1 (1:1000, Millipore, ABS787), rabbit anti-ATP5A (1:1000 ab176569, Abcam), rabbit anti-cyt C (1:1000, Abcam, ab18738) mouse anti-COX IV (1:1000 Abcam, ab14744), mouse anti- GAPDH (1:1000, Abcam, ab9484,). After PBS washes, secondary antibodies were added for 1 hour at room temperature. Secondary antibodies used were; IR dye 800CW goat anti-mouse IgG (926–32210), IR dye 800CW goat anti-rabbit IgG (926–3211), IR dye 680RD goat anti-mouse IgG (926–68070), IR dye 680RD goat anti-rabbit IgG (926–68071), all 1:5,000, LI-COR Biosciences. Membranes were imaged using an odyssey infrared imaging system (Li-COR, Biosciences) as described previously [80], and quantified using an image studio software (Li-COR). To determine final relative protein expression, band intensities were normalized to a loading control or a ponceau stain.

### Morpholino knockdown in zebrafish

Previously published *smn* morpholino was designed against the 5’ start sequence of the *smn* gene (Gene Tools LLC); 5’ CGACATCTTCTGCACCATTGGC ‘3.

An antisense MO was designed against the translational start codon of the pgk1 gene (Gene Tools LLC): 5’ TCGAAAGAGACATTTTGCCTGTGGT ‘3.

A control MO from gene tools was also used to confirm specificity of the motor axon phenotype (Gene Tools LLC).

Knockdown efficiency was quantified using western blot analysis, and normalized against a loading control.

### Oxygen consumption rate for the determination of respiratory function

Zebrafish embryos were injected with *smn* MO at the one cell stage and left to develop until 24 hpf. Oxygen consumption rate (OCR) was measured from embryos using a V17 Islet capture plate in the Seahorse Bioanalyser XFe24 (Agilent). System water was used as the media for wells, and for the preparation of drugs for injection during the run, two dechorinated embryos were placed in each well. All measurement cycles for the run were set to 2 minute mix, 90 second wait, and 3 minute measure. Prior to any drug administration 6 ‘basal’ measurements were taken, oligomycin was then added followed by 3 measurement cycles. FCCP was then added followed by another 3 measurement cycles. Finally, a mixture of antimycin and rotenone were added followed by 10 measurement cycles. Optimal doses of drugs were optimised separately for control and *smn* morphant embryo’s. Final concentrations of drugs were as follows; control oligomycin 50–75 μM, *smn* morphant oligomycin 25μM, control FCCP 20 μM, *smn* morphant FCCP 3μM. Antimycin and rotenone were added to a final concentration of 2uM for both control and *smn* morphants. To calculate basal respiration from each well, the last OCR measurement following the addition of antimycin and rotenone was subtracted from the 6^th^ basal OCR measurement. ATP linked (oligomycin sensitive) respiration was calculated by subtracting the lowest of the 3 OCR measurements following the addition of oligomycin, from the 6^th^ basal OCR measurement. Proton leak respiration was calculated by subtracting the last OCR measurement following the addition of antimycin and rotenone from the lowest OCR measurement following oligomycin. Maximal respiration was calculated by subtracting the last OCR measurement following the addition of antimycin and rotenone from the highest OCR measurement following FCCP. Spare respiratory capacity was calculated by subtracting the 6^th^ basal OCR measurement from the highest OCR measurement following FCCP. Any well, that after calculation gave rise to a negative value for their ATP linked respiration, was excluded from the analysis. One well from the control, and one well from the *smn* morphant group were excluded from this data by this criterion.

### Construct generation and in vitro transcription reaction

Overexpression constructs were generated from cDNA and ligated into an PCS2+MT expression vector. Zebrafish full-length Pgk1 was ligated using the *bamH1* restriction sites. Mouse full-length Necdin was ligated using *xho1* and *xba1* sites. Vectors were linearized and an *in vitro* transcription reaction performed using the SP6 promoter using the mMessage mMachine kit (AM1340, Ambion).

### Zebrafish rescue experiments

Single cell stage Tg(*hb9*:GFP) embryos were injected with 4ng *smn* MO in aqueous solution containing 0.05% phenol red or 4ng *smn* MO co-injected 200 ng/μl full length zebrafish *pgk1* or full length mouse 300 ng/μl *necdin* mRNA in aqueous solution and left to develop until 30 hpf for evaluation of motor axon phenotype.

### Zebrafish drug experiments

Single cell stage Tg(*hb9*:GFP) embryos were injected with 4ng *smn* MO in aqueous solution containing 0.05% phenol red and left to develop until 6 hpf before treatment with 2.5 μM terazosin, dissolved in water (Sigma-Aldrich) with untreated *smn* morphants were kept as controls. Embryos were retreated at 24 hpf with 2.5 μM terazosin and fixed at 30 hpf for evaluation of motor axon phenotype.

### Motor axon phenotype evaluation

For immunostaining, treated Tg(*hb9*:GFP) embryos were dechorinated at 30 hpf and fixed in 4% PFA overnight before being dehydrated in 100% methanol. Embryos were rehydrated and washed in PBS before being transferred into 100% acetone for 10 minutes at -20°C for permeabilisation. Embryos were washed in PBST (PBS, 1% DMSO, 1% BSA and 0.5% triton-X 100) before being blocked in PBST plus 2% Sheep serum, followed by overnight incubation of chicken anti-GFP primary antibody (1:1,000, Abcam, ab13970). Embryos were washed in PBST and then incubated in Alexa Flour 488 secondary antibody (1:500) (Jackson 703-545-155). Embryos were mounted in 80% glycerol and a z stack was imaged using a Zeiss LSMZ10 confocal microscope. The percentage of motor neurons with normal axonal outgrowth, branched axonal outgrowth and severe truncated axons was then analysed as previously described [47] (N = 20 embryos per treatment group, 12 axons per embryo (6 segments) analysed behind the yolk and shown as a percentage). The observer was blinded to the treatment.

### Immunohistochemistry

Tissue from mice was dissected and fixed in 4% paraformaldehyde in PBS for 4 hours for spinal cords, and 1 hour for sciatic nerves at 4°C before being transferred into 30% sucrose solution over-night at 4°C for cryoprotection. Tissue was embedded in OCT (1:1 OCT and 30% sucrose for sciatic nerve) and using a cryostat, where spinal cord (25 μM) and sciatic nerve (10 μM) sections were collected on polysine-coated slides (Thermo Scientific). For immunohistochemistry, sections were permeabilized in 0.3% Triton X-100 in PBS and then blocked (4% BSA, 0.3% Triton X-100 in PBS) for 30 minutes at room temperature before overnight incubation with primary antibody solution at 4°C: anti-PGK1 (Millipore, ABS787) and mouse anti-S100 (Abcam, AB7852) or mouse anti-NF heavily phosphorylated 200 (convence, SMI-31R). After PBS washes, sections were incubated with secondary antibody solution for 2 hours at room temperature: Alexa Fluor 488 donkey anti-rabbit IgG (1:500, Life Technologies, A-21206), Alexa Fluor 594 donkey anti-mouse IgG (1:500, Life Technologies A21203). Sections were counterstained with DAPI nuclei stain (1:1,000, Life Technologies, D1306) for 10 minutes before being mounted with 10% Mowiol (Polysciences). Images were taken using a Zeiss LSMZ10 confocal microscope.

### Primary neuron culture and immunocytochemistry

Primary cortical neurons and primary motor neurons were cultured as previously described [81, 82]. Briefly, timed-mated mice were sacrificed by cervical dislocation at embryonic day 13 (E13, motor neurons) or E14 (cortical neurons). For motor neurons, the ventral spinal cord was dissected, incubated in 0.05% trypsin for 15 min at 37°C and dissociated by pipetting. A motor neuron fraction was obtained by centrifugation for 15 min at 685 x *g* of the dissociated cells on a 6% optiprep gradient column. Motor neurons were pelleted by centrifugation for 10 minutes at 170 x *g* on a 4% BSA cushion and plated in glia-conditioned medium (neurobasal medium with B27 and glutamax, incubated on mixed primary glia for 24 hours) containing 5% normal horse serum and BDNF, CNTF and GDNF at 10 ng/mL. Cultures of mixed primary cortical neurons were obtained by incubating dissected embryonic cortices in 0.25% trypsin for 10 minutes at 37°C and dissociating them using a flame-polished glass pipette. Cortical neurons were seeded at 40,000 per well and motor neurons at 10,000 per well on coverslips coated with poly-D-lysine (cortical neurons) or poly-D,L-ornithine (motor neurons) and laminin. For immunohistochemistry primary cortical neurons and primary motor neurons were fixed at day 7 in 4% PFA (Electron Microscopy Sciences) with 4% sucrose for 10 minutes, permeabilised in 0.1% Triton X-100 (Sigma) for 5 minutes, washed twice in PBS and blocked in PBS containing 2.5% BSA for 30 minutes. They were subsequently incubated with anti-PGK1 (ABS787, Millipore) and mouse beta III tubulin (T8860, Sigma) in 2.5% BSA for 1 hour at RT. After 3 washes in PBS cells were incubated with Alexa Fluor 488 donkey anti-rabbit IgG (1:500, Life Technologies, A-21206) and Alexa Fluor 594 donkey anti-mouse IgG (1:500, Life Technologies A21203) for one hour. Cells were counterstained with DAPI nuclei stain (1:1,000, Life Technologies, D1306) before being mounted with 10% Mowiol.

### Statistical analysis

Data were collected and analysed using Microsoft Excel and GraphPad Prism 6 software. Individual statistical tests are described in the main text and figure legends. For all analysis, P ≤ 0.05 was considered statistically significant. All data are expressed as mean ± SEM. For all figures; NS P > 0.05, * P ≤ 0.05, ** P ≤ 0.01, *** P ≤ 0.005, **** P ≤ 0.001.

### Study approval

All experimental procedures involving animals were conducted in accordance with United Kingdom Home Office regulations, were approved by a University of Edinburgh internal ethics committee and local veterinary staff, and were performed under license from the UK Home Office (PPL 60/4569).

## Supporting information

S1 Fig Representative images of NMJ pathology in EDL, GS, and TA muscles. (A) Images of NMJs from three differentially vulnerable hindleg muscles in SMA mice, arrows show examples of partially or unoccupied endplates in vulnerable GS and TA muscles. (B) Quantification of fully occupied endplates, note the EDL muscle remained resistant throughout SMA disease progression with a high percentage of fully occupied endplates. In contrast the GS and TA muscle showed a loss of fully occupied endplates in SMA, with an increase in partially or unoccupied endplates. The TA muscle showed the largest reduction in fully occupied endplates with less than 50% at p5 in the SMA mice. Bar chart mean and s.e.m.
(TIF)

S2 Fig Quantification of axonal outgrowth in control MO injected embryos. (A) Representative confocal micrographs of primary motor neuron axons exiting the spinal cord in un-injected control (top) and control MO (bottom) in 28 hpf Tg(*hb9*:GFP) embryos. (B) Injection of a control MO at 1mM did not lead to any motor axon phenotypes, and showed the same number of normal motor axons as the un-injected controls. Bar chart (mean & s.e.m). Unpaired two-tailed student *t-test*. NS- not significant.
(TIF)

S3 Fig Knockdown of Smn via morpholino efficiency. (A) Efficiency of Smn knockdown as determined by western blot in 48hpf zebrafish. (B) knockdown was quantified and normalized to CoxIV loading control (N = 3 per group, batches of 30 pooled zebrafish embryos per lane). Bar chart (mean & s.e.m). Unpaired two-tailed student *t-test* * P<0.05.
(TIF)

S4 Fig Negative controls for PGK1 immunohistochemistry. (A) Positive staining for PGK1 in sectioned sciatic tissue. (B) Secondary only control for PGK1 staining in sectioned sciatic tissue showed no fluorescent staining.
(TIF)

S5 Fig GAPDH and PGK1 are co-expressed in the axons and growth cones of primary motor neurons. (A) GAPDH and PGK1 are expressed in axons and growth cones of primary MNs. Scale Bar = 15 μM.
(TIF)

S6 Fig Protein levels of PGK1 in early and late symptomatic SPC. (A) PGK1 levels in control littermates and SMA p5 SPC, PGK1 was significantly reduced (10%) in the SPC of early-symptomatic P5 SMA mice (B) PGK1 levels in control littermates and SMA late-symptomatic P8 SPC, PGK1 was significantly reduced (20%) in the SPC of late-symptomatic SMA mice. N = 6 per genotype. Bar chart (mean & s.e.m) Unpaired two-tailed student *t-test* * P<0.05, ** p<0.01.
(TIF)

S7 Fig Increasing the concentration of injected pgk1 MO resulted in an increase in severe motor neurons. Injection of 0.5mM resulted in a significant increase in severe motor neurons compared to controls, with 0.75mM MO resulting in an even larger number of severe motor neurons compared to controls. Bar chart (mean & s.e.m.). Unpaired two-tailed student *t-test* * P<0.05, ** p<0.01, *** p<0.001, **** p<0.0001.
(TIF)

S8 Fig Treatment with terazosin does not cause any morphological or development defects in zebrafish embryos. Representative bright field of images 28 hpf zebrafish treated with terazosin. (A) Control untreated zebrafish. (B) Treatment of 2.5 μM Terazosin and (C) Treatment of 5 μM Terazosin (TZ) from 6 hpf does not lead to any morphological or developmental delay compared to untreated zebrafish.
(TIF)
