# Supplementary figures and images for: Bioenergetic status modulates motor neuron vulnerability and pathogenesis in a zebrafish model of spinal muscular atrophy
Source: PLoS Genet. 2017 Apr 20;13(4):e1006744. doi: 10.1371/journal.pgen.1006744 (PMC5417717; doi:10.1371/journal.pgen.1006744)

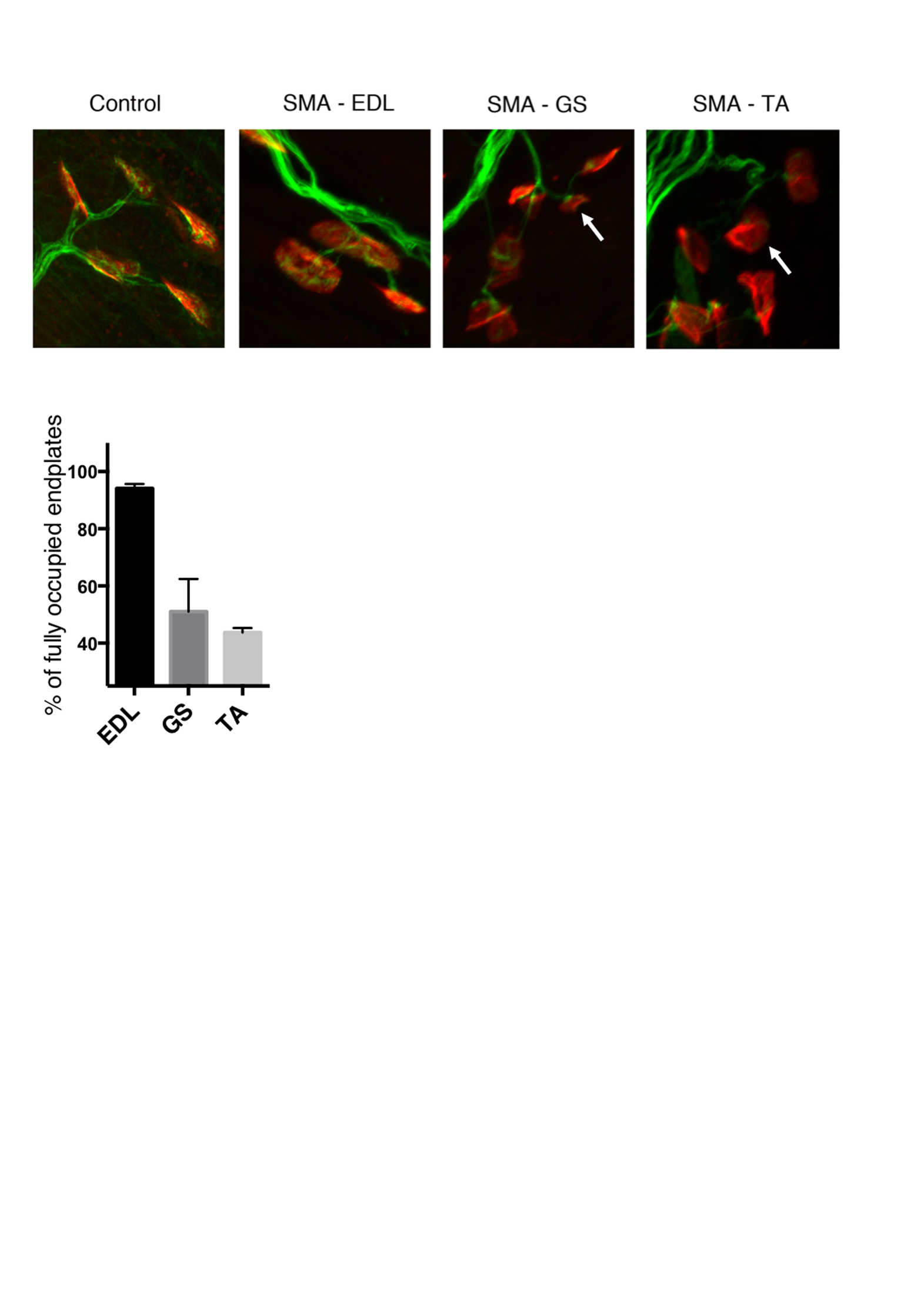

Supplement: S1 Fig — (A) Images of NMJs from three differentially vulnerable hindleg muscles in SMA mice, arrows show examples of partially or unoccupied endplates in vulnerable GS and TA muscles. (B) Quantification of fully occupied endplates, note the EDL muscle remained resistant throughout SMA disease progression with a high percentage of fully occupied endplates. In contrast the GS and TA muscle showed a loss of fully occupied endplates in SMA, with an increase in partially or unoccupied endplates. The TA muscle showed the largest reduction in fully occupied endplates with less than 50% at p5 in the SMA mice. Bar chart mean and s.e.m. (TIF) [file pgen.1006744.s001.tif]

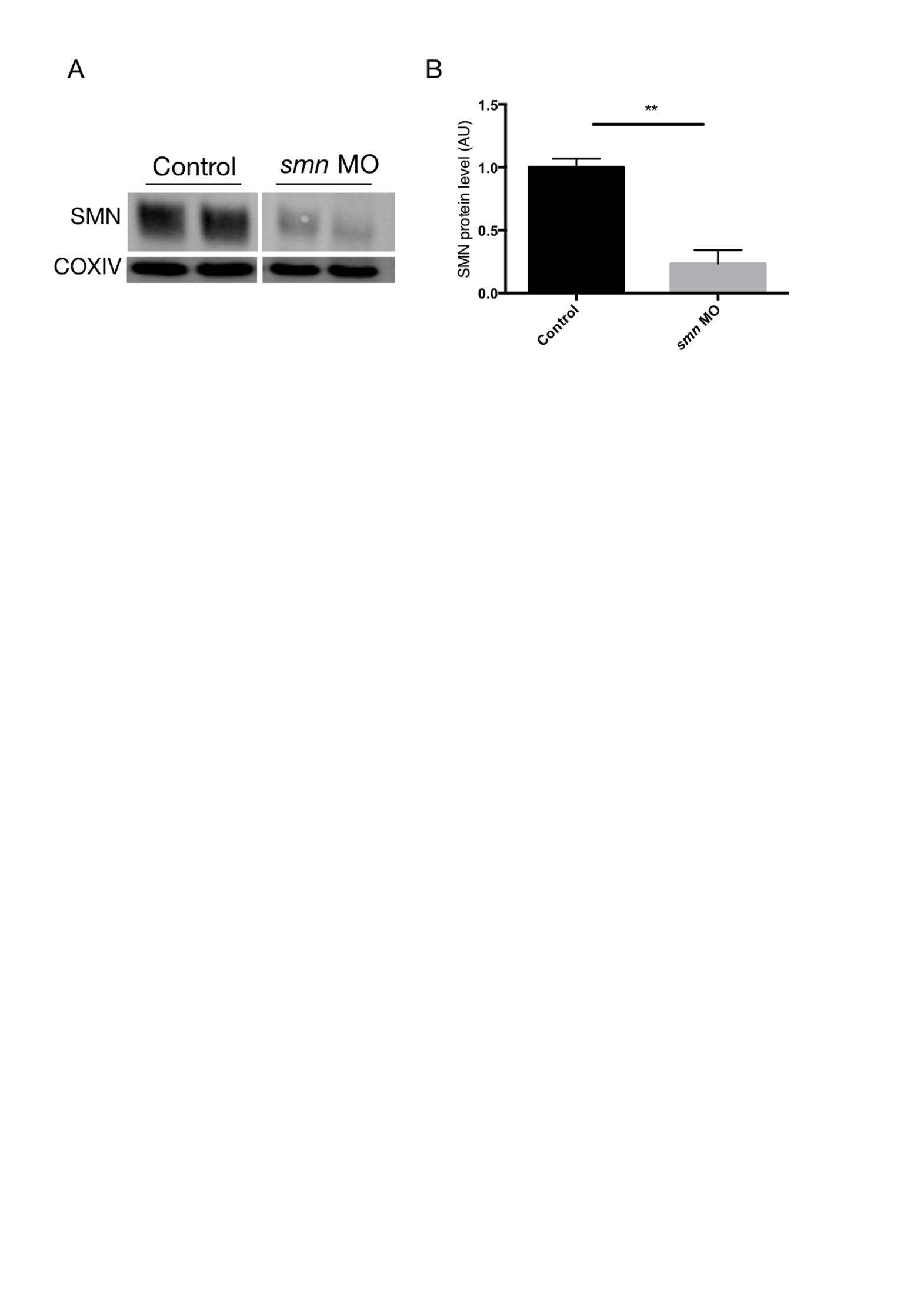

Supplement: S2 Fig — (A) Representative confocal micrographs of primary motor neuron axons exiting the spinal cord in un-injected control (top) and control MO (bottom) in 28 hpf Tg(hb9:GFP) embryos. (B) Injection of a control MO at 1mM did not lead to any motor axon phenotypes, and showed the same number of normal motor axons as the un-injected controls. Bar chart (mean & s.e.m). Unpaired two-tailed student t-test. NS- not significant. (TIF) [file pgen.1006744.s002.tif]

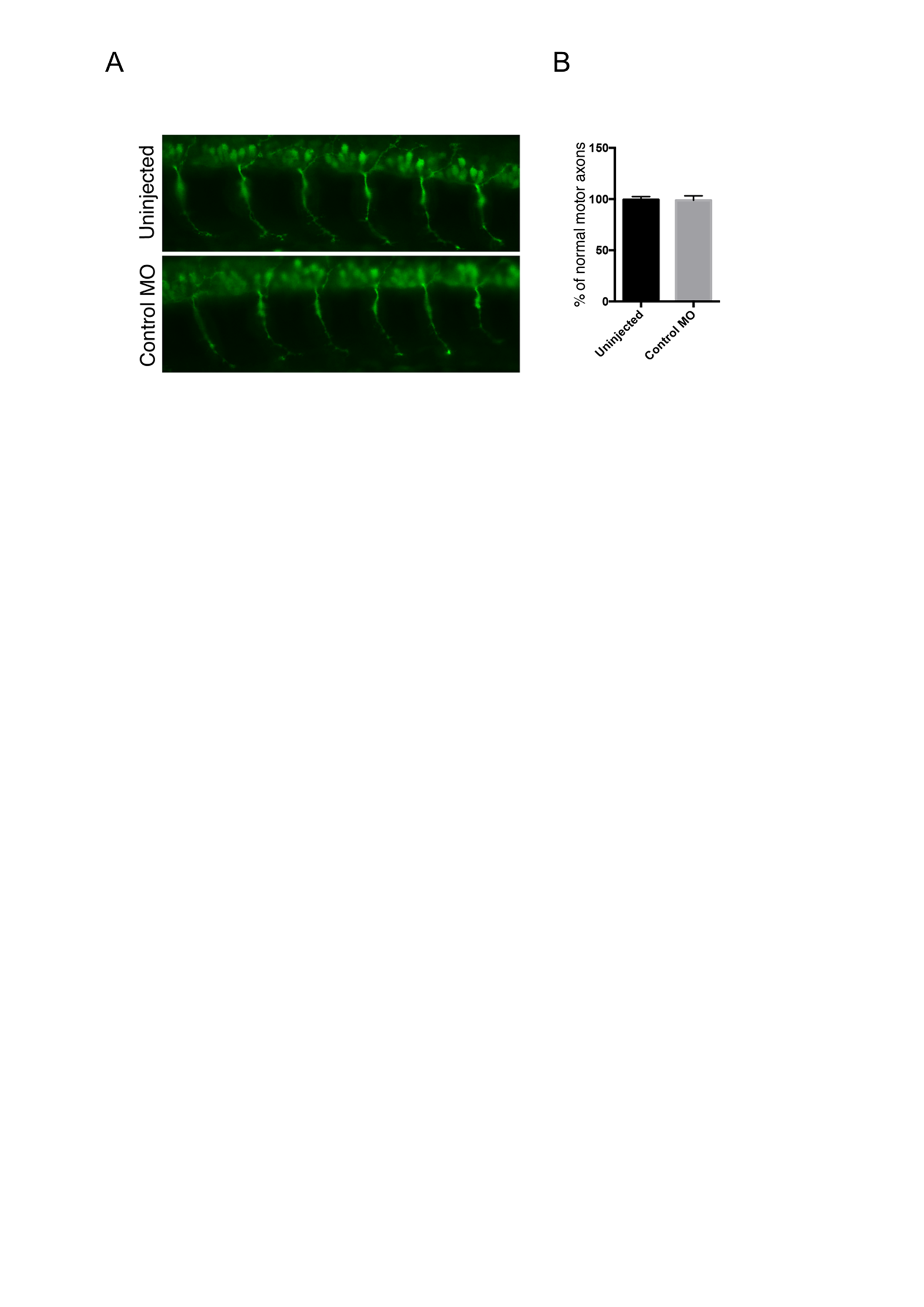

Supplement: S3 Fig — (A) Efficiency of Smn knockdown as determined by western blot in 48hpf zebrafish. (B) knockdown was quantified and normalized to CoxIV loading control (N = 3 per group, batches of 30 pooled zebrafish embryos per lane). Bar chart (mean & s.e.m). Unpaired two-tailed student t-test * P<0.05. (TIF) [file pgen.1006744.s003.tif]

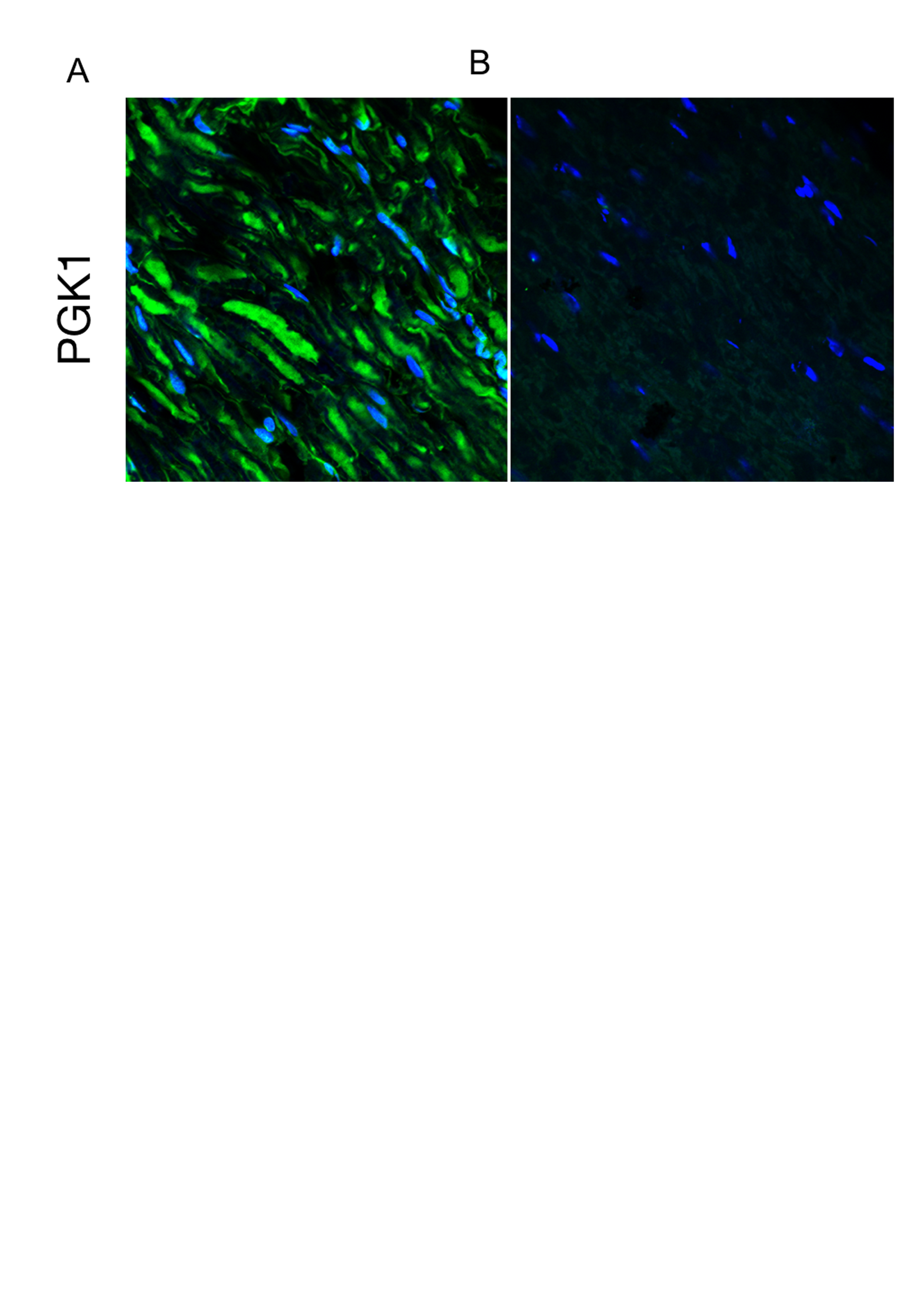

Supplement: S4 Fig — (A) Positive staining for PGK1 in sectioned sciatic tissue. (B) Secondary only control for PGK1 staining in sectioned sciatic tissue showed no fluorescent staining. (TIF) [file pgen.1006744.s004.tif]

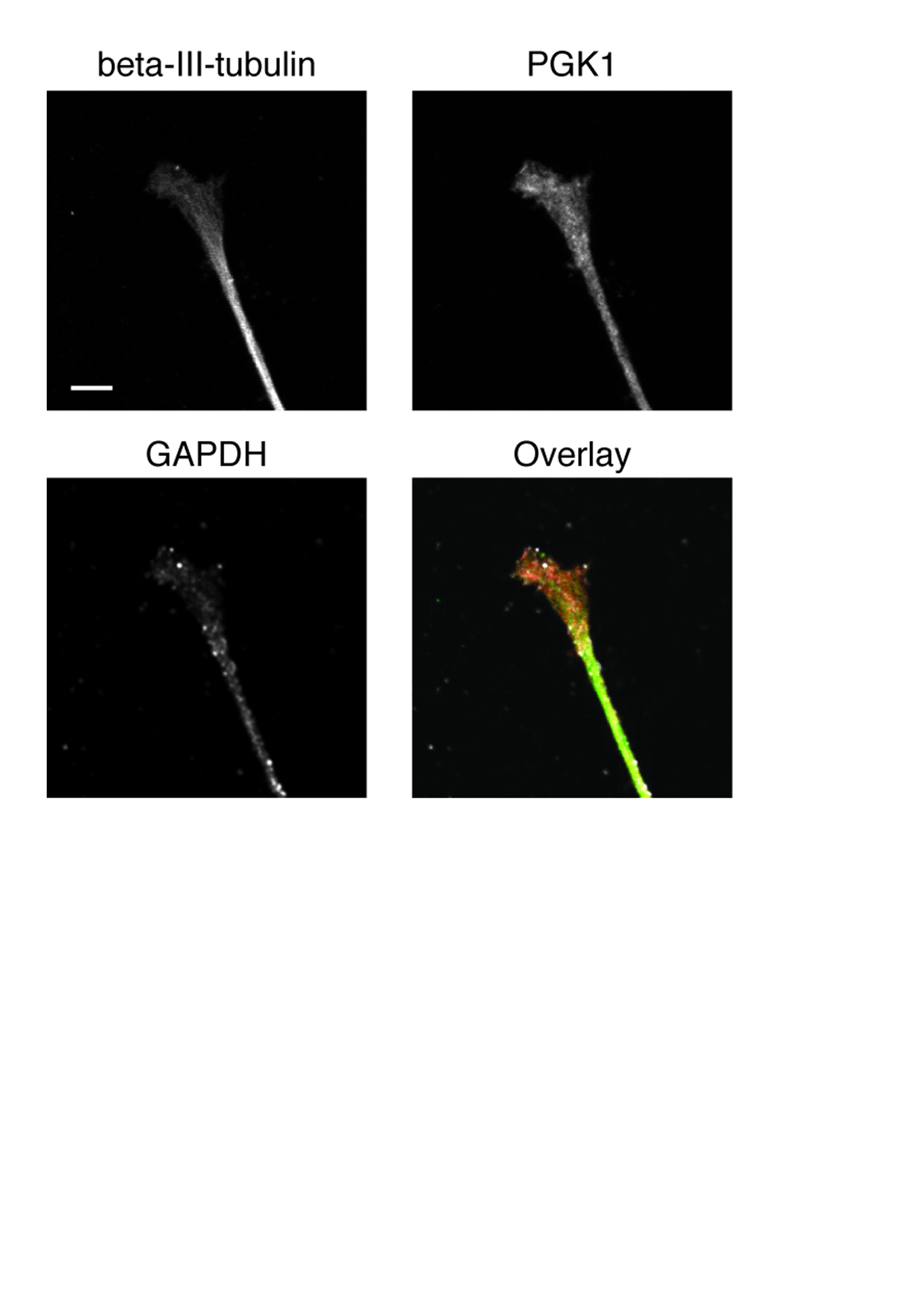

Supplement: S5 Fig — (A) GAPDH and PGK1 are expressed in axons and growth cones of primary MNs. Scale Bar = 15 μM. (TIF) [file pgen.1006744.s005.tif]

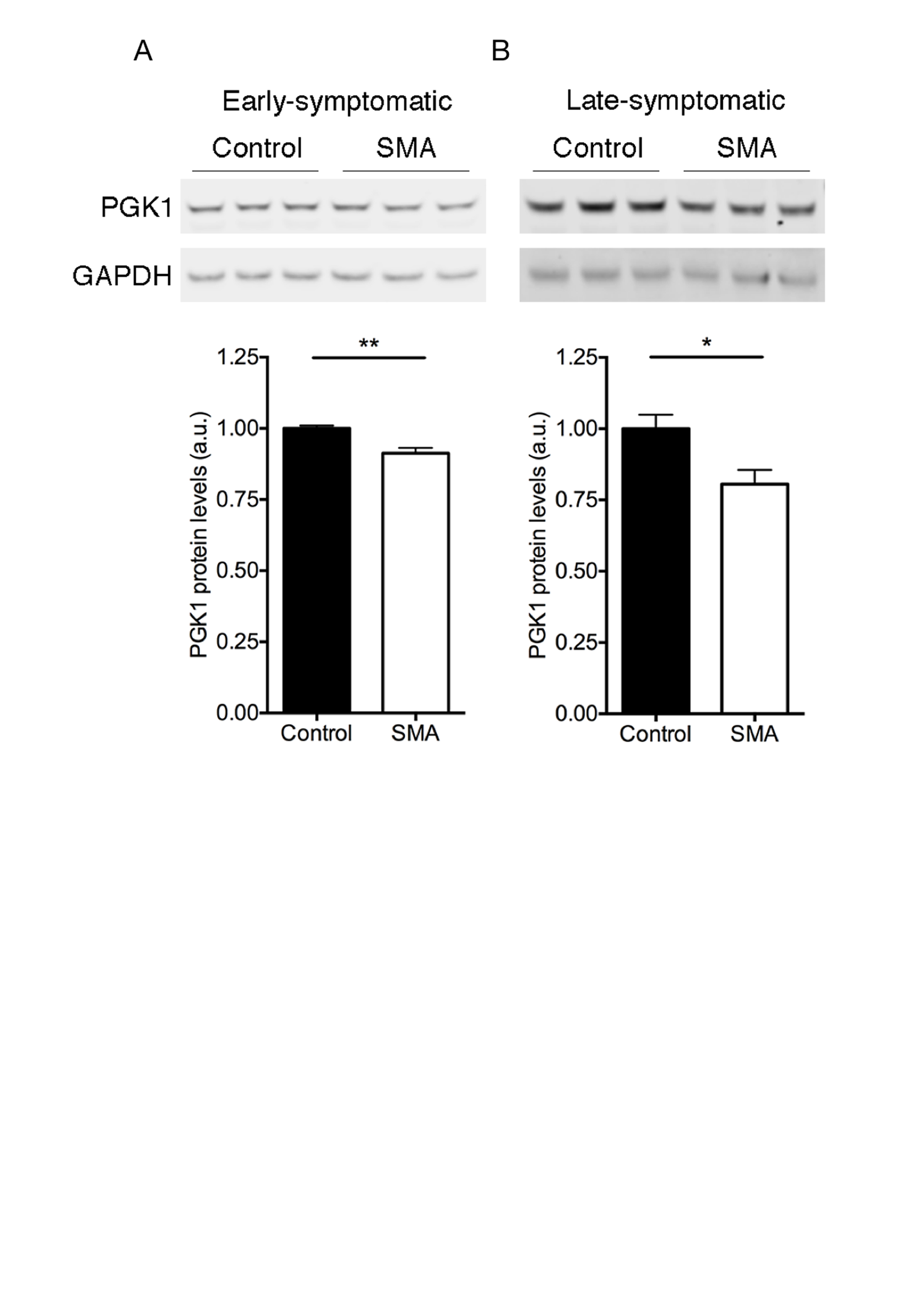

Supplement: S6 Fig — (A) PGK1 levels in control littermates and SMA p5 SPC, PGK1 was significantly reduced (10%) in the SPC of early-symptomatic P5 SMA mice (B) PGK1 levels in control littermates and SMA late-symptomatic P8 SPC, PGK1 was significantly reduced (20%) in the SPC of late-symptomatic SMA mice. N = 6 per genotype. Bar chart (mean & s.e.m) Unpaired two-tailed student t-test * P<0.05, ** p<0.01. (TIF) [file pgen.1006744.s006.tif]

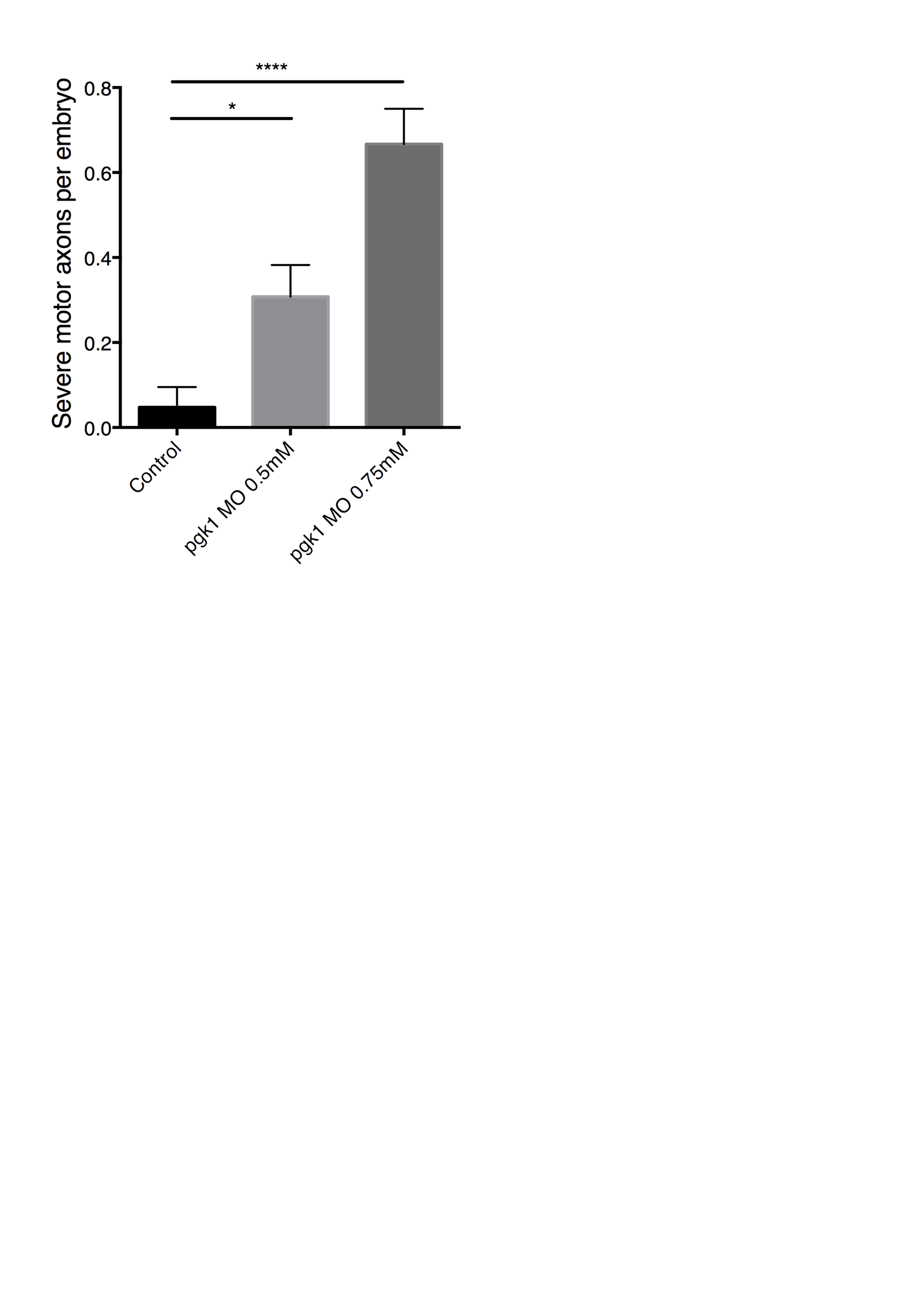

Supplement: S7 Fig — Injection of 0.5mM resulted in a significant increase in severe motor neurons compared to controls, with 0.75mM MO resulting in an even larger number of severe motor neurons compared to controls. Bar chart (mean & s.e.m.). Unpaired two-tailed student t-test * P<0.05, ** p<0.01, *** p<0.001, **** p<0.0001. (TIF) [file pgen.1006744.s007.tif]

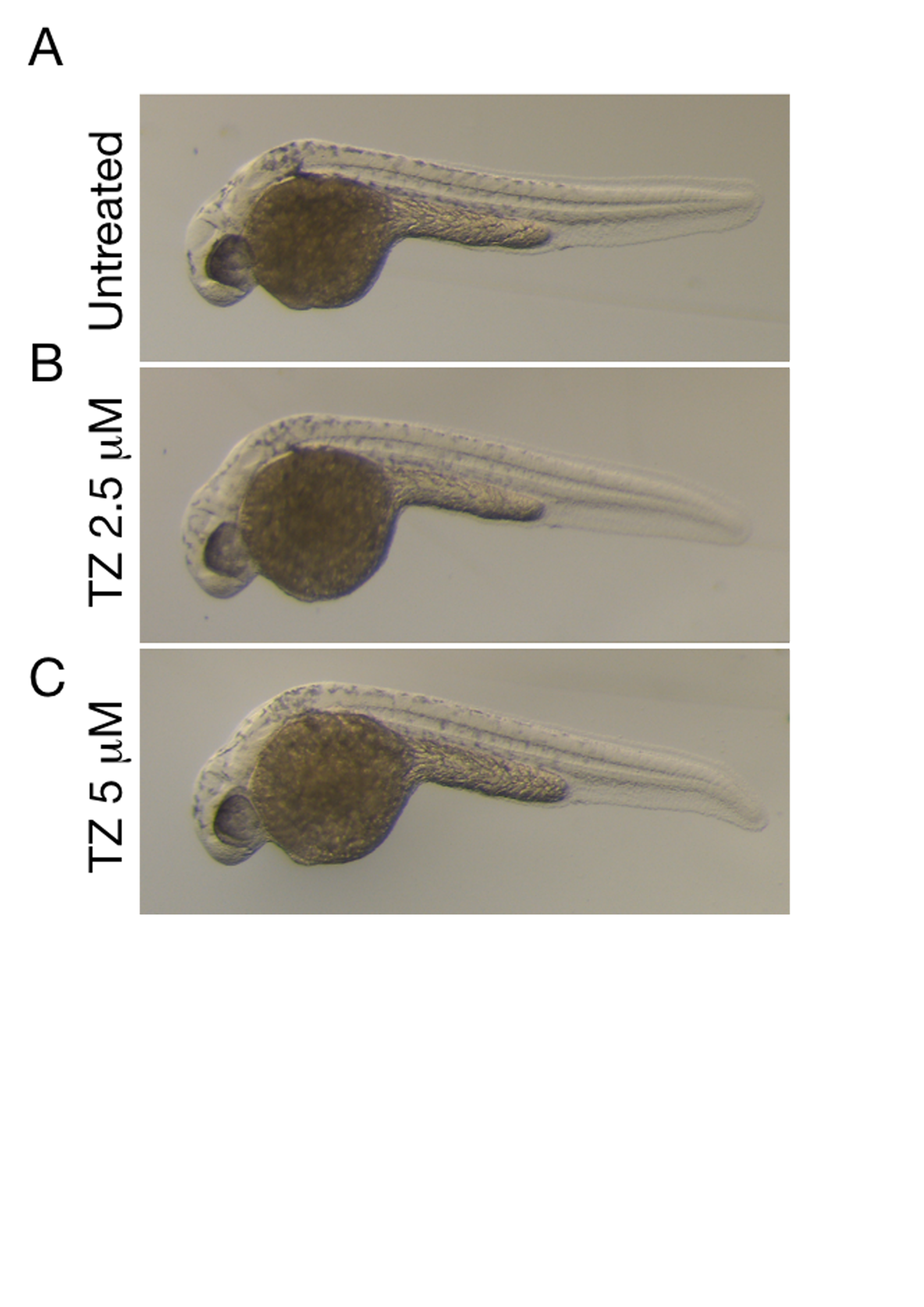

Supplement: S8 Fig — Representative bright field of images 28 hpf zebrafish treated with terazosin. (A) Control untreated zebrafish. (B) Treatment of 2.5 μM Terazosin and (C) Treatment of 5 μM Terazosin (TZ) from 6 hpf does not lead to any morphological or developmental delay compared to untreated zebrafish. (TIF) [file pgen.1006744.s008.tif]
